# FGF21-FGFR1 signaling protects against cardiac hypertrophy by regulating PINK1-mediated mitophagy pathway

**DOI:** 10.1016/j.jare.2025.10.053

**Published:** 2025-10-29

**Authors:** Lei Chen, Lingxin Zheng, Yuan Qin, Bilin Liu, Yejing Zheng, Xiaojian Tong, Mengting Dai, Guohua Gong

**Affiliations:** a Key Laboratory of Laboratory Medicine, Ministry of Education, School of Laboratory Medicine and Life Sciences, Wenzhou Medical University, Wenzhou 325035, China; b Institute for Regenerative Medicine, State Key Laboratory of Cardiology and Medical Innovation Center, Shanghai East Hospital, School of Life Sciences and Technology, Tongji University, Shanghai 200092, China; c Department of Blood Transfusion, The First Affiliated Hospital (Southwest Hospital) of Army Medical University, Chongqing 400038, China; d The First School of Clinical Medicine, Nanjing University of Chinese Medicine, Nanjing 210023, China

**Keywords:** FGF21, PINK1, FGFR1, Cardiac hypertrophy, Mitophagy

## Abstract

**Introduction:**

Cardiac hypertrophy is an independent risk factor and the primary predictor of heart failure (HF). Mitochondria are crucial for the shift from hypertrophy to heart failure. The expression of fibroblast growth factor 21 (FGF21), a cardioprotective factor, is increased in patients with cardiac hypertrophy but fails to prevent heart failure. Additionally, the molecular mechanism through which FGF21 exerts its beneficial effects on hypertrophic myocardial mitochondria remains unclear.

**Objectives:**

Our study investigated the effect of FGF21 on cardiac hypertrophy, elucidating its mechanism of action through the enhancement of mitophagy-mediated cardioprotection.

**Methods:**

A transverse aortic constriction (TAC) model and a phenylephrine (PE) model were applied to explore the effect and mechanism of FGF21. P62-mediated mitophagy inducer (PMI) and rapamycin (Rapa) were used to confirm that FGF21-regulated mitophagy under overload pressure conditions.

**Results:**

FGF21 knockout markedly exacerbated TAC-induced cardiac function damage, mitochondrial damage, and mitophagy impairment. In vitro, FGF21 knockdown aggravated PE-induced cardiomyocyte hypertrophy and mitophagy dysfunction. FGF21 treatment promoted mitophagy in the TAC and PE models, but this effect was abolished in the absence of PTEN-induced putative kinase 1 (PINK1). The increase in PINK1 expression induced by Rapa can rescue impaired cardiac function and mitophagy impairment in FGF21-deficient TAC mice. Similarly, PMI enhances mitophagy, which inhibits damage to cardiac functions. A further study revealed that the expression of fibroblast growth factor receptor 1 (FGFR1) and FGF21 was opposite in heart failure. Knockdown of FGFR1 inhibited FGF21-mediated mitophagy.

**Conclusion:**

FGF21 promotes PINK1-mediated mitophagy to attenuate cardiac hypertrophy, and mismatched FGFR1 expression may hamper the beneficial effect of FGF21 on cardiac hypertrophy.

## Highlights

• FGF21 is a cardioprotective factor in hypertrophy.
• FGF21 promotes PINK1-mediated mitophagy to attenuate cardiac hypertrophy.
• Enhancing mitophagy prevents fgf21 KO mice from TAC-induced hypertrophy.
• FGFR1 and FGF21 are mismatched in expression during heart failure development.

## Graphical abstract

**Figure:**
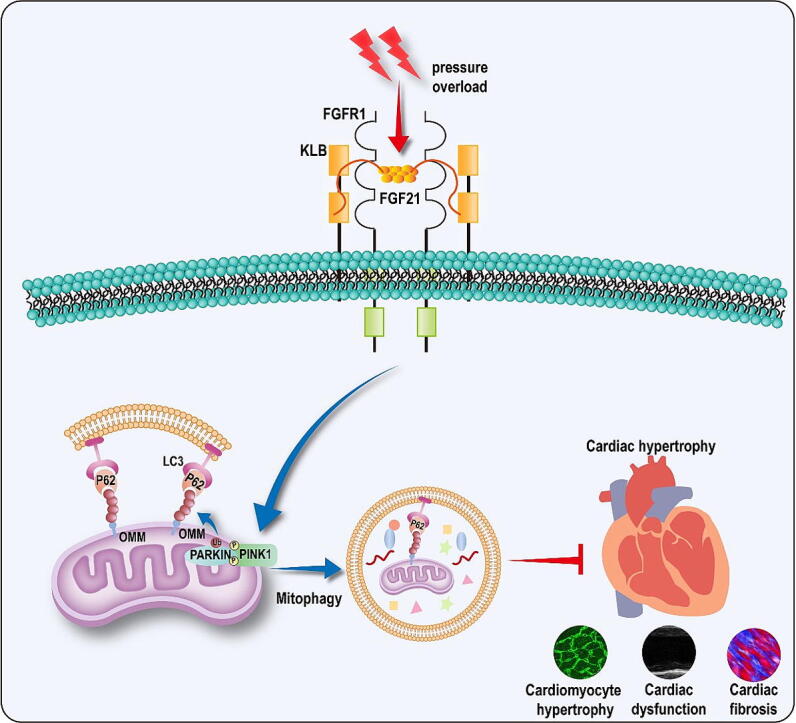

## Introduction

Cardiac hypertrophy progresses to heart failure (HF) in response to diverse cardiac stresses such as pressure or volume overload, and hypoxia. This process is characterized by an increase in cardiomyocyte size and volume, myocardial fibrosis, and the generation of aberrant gene expression, as well as a decrease in cardiac function [1,2]. Cardiac hypertrophy is an independent risk factor for heart failure and is a significant predictor of cardiovascular morbidity and mortality [3]. While numerous significant molecules and signaling pathways have been discovered in the onset and progression of cardiac hypertrophy, the pathogenesis of this condition is diverse and complex, and its molecular mechanisms remain incompletely characterized and understood. Therefore, further research is necessary to explore this new strategy for treating cardiac hypertrophy and heart failure in more detail.

Mitochondria constitute approximately 40 % of the volume of adult cardiomyocytes and are responsible for generating approximately 90 % of ATP [[4], [5], [6], [7]], which is vital for maintaining cardiac function. Mitochondrial quality control (MQC), which includes mitochondrial biogenesis, mitochondrial dynamics, and mitophagy, is essential for preserving mitochondrial homeostasis. Under conditions of pressure overload, mitochondria are impaired, and generate reactive oxygen species (ROS) [[8], [9], [10], [11]], release cytochrome *c* [12], and produce other harmful molecules. This results in damage to cardiomyocytes and further causes functional disorders of the heart. Thus, the elimination of impaired and dysfunctional mitochondria as well as the promotion of mitochondrial turnover is crucial for preserving cardiomyocyte function. Mitophagy, a selective autophagy process, eliminates dysfunctional and damaged mitochondria to maintain their proper quality and quantity [13,14]. Mitophagy is dysregulated in cardiac hypertrophy or heart failure induced by transverse aortic constriction (TAC). Mitophagy serves as a critical regulator of the pathogenesis of cardiac hypertrophy. Mice lacking key mitophagy regulators, such as PARKIN or PTEN-induced putative kinase 1 (PINK1), display pathological hypertrophy with age [15]. Enhancing mitophagy to remove dysfunctional mitochondria rescues mitochondrial quality control, which is beneficial for the maintenance of cardiomyocyte function [16]. Pharmacological treatment or genetic methods can rescue disordered mitophagy, improving cardiac function and inhibiting cardiac remodeling.

Fibroblast growth factor 21 (FGF21), a secreted factor, can be expressed in the heart and subsequently released [17,18]. Studies have shown that while the level of FGF21 is low in the heart, this level increases in cases of cardiac hypertrophy and heart failure [17,19,20]. A recent study revealed that FGF21 can inhibit cardiomyocyte apoptosis in response to myocardial ischemia, thereby improving cardiac function [21]. Additionally, FGF21 treatment has been shown to attenuate inflammation and oxidative stress in cardiac hypertrophy [22]. FGF21 can repress ischemia/reperfusion injury in the heart by enhancing autophagy [23]. These findings suggest that FGF21 serves as a cardioprotective factor. Previous reports have revealed that FGF21 regulates mitophagy signaling pathways in muscle [24]. However, the specific roles of FGF21 in mitophagy and mitochondrial function, particularly in the context of cardiac hypertrophy, remain unclear.

Our study investigated the molecular mechanism of FGF21 in cardiac hypertrophy in vivo and in vitro. The data revealed that a decrease in FGF21 aggravated TAC- or phenylephrine (PE)-mediated cardiomyocyte hypertrophy, mitochondrial dysfunction, and mitophagy impairment. Mechanistically, we found that PINK1 knockdown abolished the enhancing effect of FGF21 on mitophagy in the TAC and PE models, suggesting that FGF21 promoted mitophagy through the regulation of PINK1. Rapamycin (Rapa) and a P62-mediated mitophagy inducer (PMI) enhanced mitophagy in TAC-mediated *Fgf21^-/-^* mice, which improved cardiac function and significantly inhibited the progression of cardiac hypertrophy. Further experiments revealed that knockdown of fibroblast growth factor receptor 1 (FGFR1) abolished the enhancing effect of FGF21 on mitophagy. A decrease in FGFR1 expression might prevent the protective effect of FGF21 in heart failure.

## Materials and methods

**Antibodies and reagents**.

The following commercial antibodies were used: FGF21 (abclonal, A3908), ANP (santa cruz, sc-515701), BNP (santa cruz, sc-271185), PGC-1α (santa cruz, sc-518025), Mfn2 (proteintech, 12186–1-AP), Drp1 (abcam, Ab56788), PINK1 rabbit mAb (abcam, Ab75487), PARKIN rabbit mAb (santa cruz, sc-32282), SQSTM1/P62 (santa cruz, sc-48402), LC3A/B (abcam, Ab128025), Beclin1 (abcam, Ab114071), VDAC (abcam, Ab15895), FUNDC1 (CST, E2F4T), BNIP3L/NIX (CST, 12396S), GAPDH (sangon, D110016); total OXPHOS WB antibody cocktail (abcam, ab110413). Phenylephrine (PE, #HY-B0769) and FGF21 protein(#HY-P70473), Efruxifermin (#HY-P99930) and rapamycin (Rapa, #HY-10219) were purchased from MCE. P62-mediated mitophagy inducer (PMI, #T13809).

## Ethics statement

All experiments involving animals were conducted according to the ethical policies and procedures approved by the Laboratory Animal Management and Ethics Committee of Wenzhou Medical University (Approval Number: xmsq2022-1323).

## Transverse aortic constriction model

We purchased *Fgf21^-/-^* mice and *Pink1^-/-^* mice from cyagen. We purchased C57BL/6 mice from SLAC. The TAC surgery was used to induce, which was performed as follows. Briefly, 2 % isoflurane was applied for anaesthetizing mice (RWD, #R510-22–10). Then we exposed and isolated the aortic arch. Next, we constricted the fraction of the aorta localized between the brachiocephalic artery and the left common carotid artery with a 27-gauge needle and a 6.0 silk thread. Finally, we sutured the incision. Mice were placed in a warm environment until they woke up, and then were returned to their cages. Sham group mice underwent the same procedure without the constriction of the aortic arch.

One week after TAC, WT or *Pink1^-/-^* mice were injected subcutaneously with saline or efruxifermin at 1 mg/kg [25,26] weekly for four weeks. Three weeks after TAC, *Fgf21^-/-^* mice were injected intraperitoneally with solvent or Rapa at 2 mg/kg [27] for two weeks. One week after TAC, Solvent or PMI (10 mg/kg) (The laboratory-optimized experimental concentrations are not shown (data not shown)) was intraperitoneally injected into *Fgf21*^-/-^ mice daily for two weeks.

## Echocardiographic examination

Echocardiography (Visual Sonics Vevo3100 system) was used to assess the cardiac function after TAC at four weeks or five weeks. Mice were inhalation anesthetized with a mixture of 2 % isoflurane and oxygen, and then were placed on a heated platform, whose heart rate was maintained at 450–500 beats per minute before transthoracic echocardiography detection. The left ventricle and the aortic outflow tract were observed under B-mode, which guided the probe to find the maximum cross-section of the left ventricle. M−mode echocardiographic images were recorded. M−mode analysis was used to calculate left ventricular ejection fraction (LVEF) and left ventricular fractional shortening (LVFS).

## RNA isolation and real-time PCR

HiPure Total RNA Mini Kit (Magen, #R4111-03) was used to extract total RNA. The cDNA was prepared according to the instructions of the reverse transcript kit (Vazyme, #R323-01). The primers were synthesized by Sangon, as shown in Table S1.Target gene expression was amplified using SYBR Mix (Vazyme, #Q711-02) and detected using a real-time PCR instrument (Bio-Rad). Data were normalized to loading control (GAPDH) for each experiment.

## Western blot

Heart tissue proteins were extracted using a lysis buffer (1 M HEPES, pH 7.2, Sucrose, 1 M MgCl2, 100 mM DTT plus protease inhibitor). The protein in the cells was extracted using M−PER Mammalian Protein Extraction Reagent (Thermo Fisher Scientific, #78505). The concentration of protein was measured with the Pierce BCA Protein Assay Kit (Thermo Scientific, #23225). The proteins were separated using SDS-PAGE and transferred onto a PVDF membrane (Millipore, #IPFL00005). The membrane was incubated with primary antibodies and subsequent HRP-conjugated secondary antibodies. The target bands were detected with ECL chemiluminescence reagent (Beyotime, #P0018FM). Protein expression was quantified using ImageJ software (National Institute of Health, Version 1.8).

## Histological staining

The heart tissues were isolated and fixed in a 4 % paraformaldehyde solution (Sangon, #E672002) at 4 °C for 24 h, after which they were dehydrated and embedded in paraffin. The embedded hearts were sliced into 5 μm sections. The sections were deparaffinated and then stained with hematoxylin-eosin (HE) regent (Sangon, #E607318), Masson Trichrome kit (Sigma-Aldrich, # HT15), or wheat germ agglutinin (Invitrogen, #W11261) regent according to the manufacturer's instructions. HE and Masson images were captured using a DMi8 microscope (Leica). The pictures of WGA were taken by confocal microscopy (LSM880, Zeiss), which were collected from at least 5 independent fields at each section. The fibrosis area of each slice was measured using ImageJ software. The cross-sectional area of each cardiomyocyte in each slice was also measured using ImageJ software.

## Transmission electron microscopy

The heart tissues were harvested, cut into pieces measuring approximately 1 mm^3^, and fixed immediately in 2.5 % glutaraldehyde at 4°C overnight. The samples were then washed three times with 0.1 M PBS buffer, post-fixed with 1 % OsO_4_ for 1 h, washed three times again with 0.1 M PBS buffer, counterstained with uranyl acetate for 1.5 h, dehydrated in acetone gradients, and embedded in araldite resin. Ultrathin sections were cut. The pictures were acquired using a Biology Transmission Electron Microscope (JEOL JEM-1010, JEOL). ImageJ was used to measure the mitochondrial area and autophagosome number.

## ATP measurement

Enhanced ATP Assay Kit (Beyotime, #S0027) was used to detect the ATP content of heart tissue or cells. Briefly, cardiac tissue or cells were lysed with ATP lysis buffer. After centrifugation of the lysis mixture at 12,000*g* for 5 min at 4 °C, the supernatant was collected. 20 µl supernatant or different standard substances were then added into 100 µl ATP detection working solution in an untransparent 96-well plate, respectively. A microplate reader (Infinite M 200 PRO, Tecan) was used to measure fluorescence intensity. The concentration of proteins was measured with the Pierce BCA Protein Assay Kit. Data were normalized according to the standard curve and protein concentration. Data are presented in nmol/mg protein.

## Mitochondria isolation

Mitochondria of heart tissues were isolated using lysis buffer (1 M HEPES, pH 7.2, Sucrose, 1 M MgCl2, 100 mM DTT plus protease inhibitor). Heart tissues were homogenized and centrifuged at 600 × g for 10 min at 4 °C. The supernatant was then collected and centrifuged at 3500 × g for 10 min at 4 °C. The pellet (mitochondrial protein) was resuspended in homogenizing buffer and stored at −80 °C. The supernatant is then centrifuged at 12,000 × g for 10 min at 4 °C. The supernatant is the cytoplasmic protein.

## Neonatal rat cardiomyocytes isolation, culture, transfection, and treatment

Primary neonatal rat cardiomyocytes (NRCMs) were isolated from 1 to 2-day-old SD rats. Briefly, the hearts were removed and cut into small pieces measuring 1 mm^3^ in an ice-cold PBS solution. The small cardiac tissue was digested with 0.125 % trypsin (Thermo Fisher Scientific, #25300054) and 0.14 mg/ml type II collagenase (Worthington, #LS004174) at 37℃ for 6 min. The cell suspension was filtered through a 100-mesh cell sieve. The cell suspension was centrifuged at 1500 rpm for 6 min. The supernatant was discarded, and the cell precipitation was resuspended with DMEM medium. The cells were cultured at 37℃, 5 %CO_2_ for 90 min. NRCMs were isolated using differential adhesion to remove cardiac fibroblasts. Purified cardiomyocytes were cultured in laminin-coated culture dishes with DMEM supplemented with 10 % FBS and 1 % PS at 37℃, 5 %CO_2_.

NRCMs were treated with 100 μM PE for 24 h. Transfection of target-specific siRNAs and negative control (NC) siRNAs (GenePharma) was performed according to the manufacturer's protocol. The following siRNA sequences were used: FGF21 siRNA: sense 5′-CGACAGAGGUAUCUCUACACA-3′ antisense 5′-UGUAGAGAUACCU CUGUCGGA-3′, PINK1 siRNA: sense 5′-GCCCAGAUGUCGUCUCAAA-3′ antisense 5′-UUUGAGACGACAUCUGGGC-3′. FGFR1 siRNA: sense 5′-TGAAG ACTGCTGGAGTTAATA-3′, antisense 5′-TATTAACTCCAGCAGTCTTCA-3′, FGFR3 siRNA: sense 5′-CAGGTGTCCTTGGAGTCTAAT-3′, antisense 5′-ATTAGA CTCCAAGGA CACCTG-3′.

TMRM staining of living cells.

We isolated adult mouse cardiomyocytes according to our previous study [28]. The mouse heart was removed four weeks after TAC and perfused using a Langendorff system with Ca2^+^-free KHB. Then, the heart was digest with collagenase II solution. Finally, the atrium and aorta were removed, and the ventricular tissues were digested with 0.05 % Trypsin-EDTA solution three times. The isolated cardiomyocytes were plated in mouse laminin pre-coated culture dishes. After 24 h of culture in a 5 % CO2 incubator at 37 °C, cardiomyocytes were stained with 50 nmol/L TMRM (Thermo Fisher Scientific, #T668) for 30 min.

## ROS measurement

NRCMs were cultured in a 96-well plate. After transfection and drug administration, total ROS was measured by H_2_DCFDA regent (TargetMol, #T15458). Mitochondrial superoxide production was evaluated using MitoSOX™ Red mitochondrial superoxide indicator (Thermo Fisher Scientific, M36008). Briefly, cultured cells were stained with 10 μM H_2_DCFDA reagent or 5 μM MitoSOX™ at 37 °C for 30 min. Cells were washed with Warm HBSS three times.

## Mitophagy vacuoles assay

NRCMs were cultured on the slide in DMEM with 10 % FBS and 1 % PS. siRNA and NC were transfected into NRCMs. Then, cells were infected with Ad-EGFP-LC3 (HanBio Technology Co. Ltd.). The next day, cells were treated with or without PE or PE + FGF21 protein. After drug administration, NRCMs were incubated in MitoTracker® Probes (Thermo Fisher Scientific, #M7513) for 30 min. Confocal microscopy images were captured from random fields.

## Mitochondrial and lysosome staining of living cells

Mitochondria and lysosomes were detected using MitoTracker® Probes (Thermo Fisher Scientific, #M7514) and LysoTracker® Probes (Thermo Fisher Scientific, #L7528). NRCMs were cultured on a slide in DMEM with 10 % FBS and 1 % PS. siRNA and NC were transfected into NRCMs. After 24 h, cells were treated with or without PE or PE + FGF21 protein. After the drug treatments, NRCMs were incubated in MitoTracker® Probes and LysoTracker® probes for 30 min. Confocal microscopy images were captured from random fields.

## Statistics

Results are presented as means ± SEM. Data were analyzed by one-way ANOVA or two-way ANOVA, followed by Dunnett's or Bonferroni post-hoc tests as appropriate. Statistical analysis was performed using GraphPad Prism9 (GraphPad Software Inc., San Diego, CA, USA).

## Results

**FGF21 deficiency promotes TAC-induced cardiac hypertrophy and eventually causes heart failure in mice**.

**Fig. 1:**
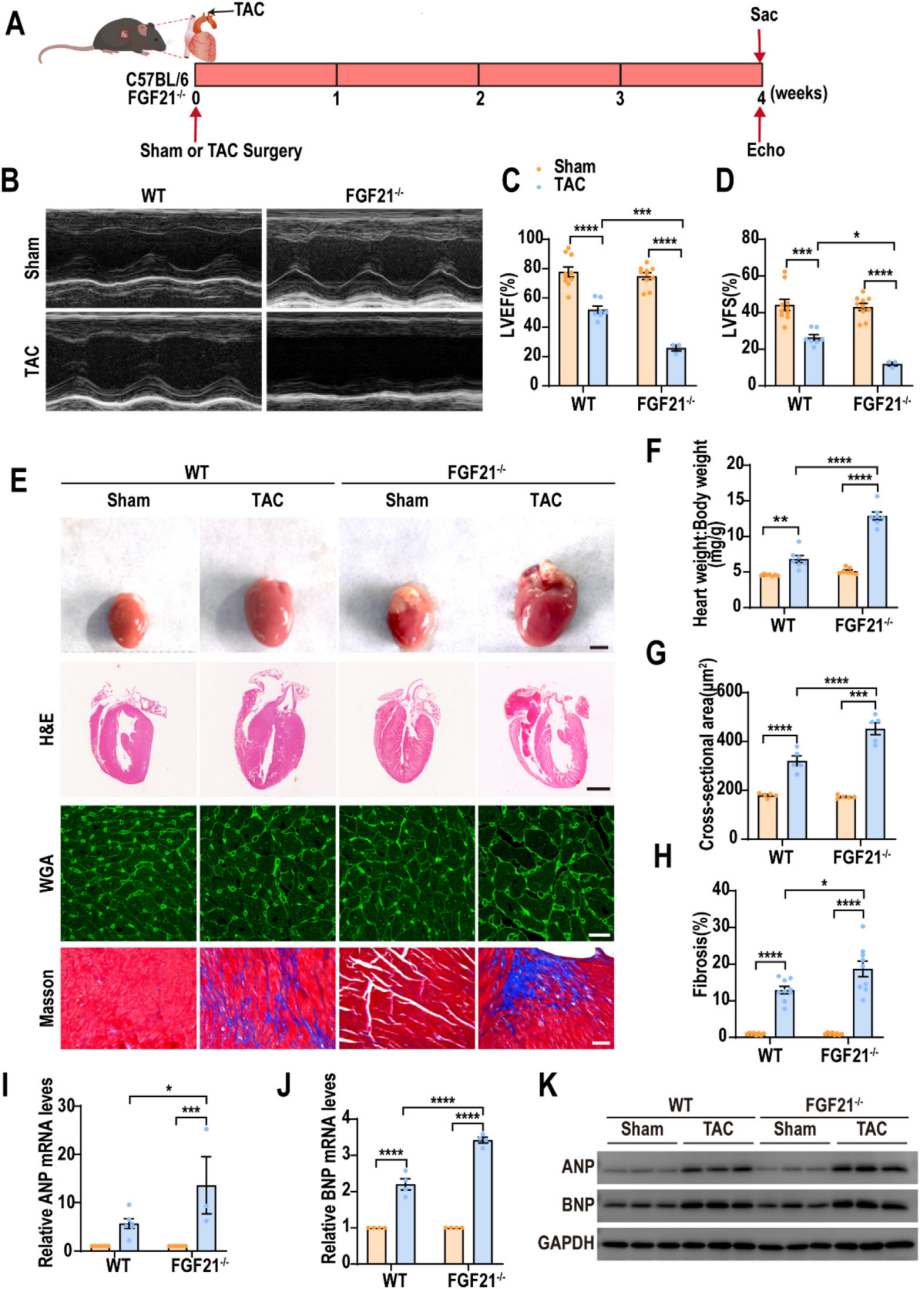
**Deletion of FGF21 promotes the onset of cardiac hypertrophy and eventually causes heart failure.** A. Schematic outline of animal experimental procedures: ten weeks WT and *Fgf21^-/-^* mice were subjected to sham or TAC operation for four weeks. B. Representative M−mode echocardiography from each group in mice. C. Measurement of LVEF in the indicated groups. D. Measurement of LVFS in the indicated groups. E. Gross hearts, scale bar = 2 mm and representative image of HE staining, scale bar = 2 mm (n = 7), representative image of WGA immunofluorescent staining (n = 5), scale bar = 20 µm, representative images of Masson's Trichrome staining (n = 7, 9), scale bar = 100 µm. F. Ratio of HW to BW. G. Quantitative analysis of cardiomyocyte cross-sectional area for each group. H. Quantitative analysis of interstitial fibrosis for each group. I, J. Relative ANP and BNP mRNA levels. K. ANP and BNP protein levels in the indicated groups (n = 4). All data represent mean ± SEM, and statistical significance was measured using the two-way ANOVA with the Bonferroni post-hoc test.

Evidence has shown that the level of FGF21 is elevated in cardiac hypertrophy and heart failure and that it protects against cardiac hypertrophy [17]. The roles and molecular mechanisms of FGF21 under these conditions are still unclear. Therefore, to investigate the underlying role of FGF21 in cardiac hypertrophy and heart failure, we used FGF21 knockout mice via the CRISPR/Cas9 method (Fig. S1A). Consistent with previous reports, the body length and appearance of the *Fgf21^-/^***^-^** mice were normal at ten weeks (Fig. S1B). The weights of the heart were unchanged (Fig. S1C). In addition, the volume of cardiomyocytes was unchanged (Fig. S1D). The levels of markers of heart failure, including atrial natriuretic peptide (ANP) and B-type natriuretic peptide (BNP), and markers of cardiac hypertrophy, such as myosin heavy chain 7 (MYH7), were not elevated (Fig. S1E–G), indicating that FGF21 knockout does not cause distinct heart damage under normal conditions. To investigate the effect of FGF21 under pressure overload conditions, wild-type (WT) and *Fgf21^-/-^* mice were used for TAC-induced cardiac hypertrophy (Fig. 1A). We performed an echocardiography (Echo) assay four weeks after TAC. Functionally, compared with those in the hearts of WT mice, the LVEF and LVFS in the hearts of *Fgf21^-/-^* mice decreased after TAC (Fig. 1, Fig. 1). Heart volume, the ratio of heart weight to body weight (HW/BW), and the cross-sectional area of cardiomyocytes significantly increased in both WT and *Fgf21^-/-^* mice after TAC (Fig. 1, Fig. 1). The increase in these parameters was more pronounced in the *Fgf21^-/-^* mice than in the WT mice after TAC (Fig. 1, Fig. 1). HE staining revealed that the left ventricular wall thickened in WT mice after TAC. However, after TAC, the left ventricular wall thinned, and the chamber enlarged in the *Fgf21^-/-^* mice (Fig. 1E). Consistent pressure overload induces cardiac remodeling, which also increases the levels of collagens in the interstitial regions of the myocardium [29]. Compared with WT mice, TAC surgery-mediated overload pressure for four weeks contributed to prominent cardiac interstitial fibrosis in the *Fgf21^-/-^* mice after TAC (Fig. 1, Fig. 1). At the molecular level, a striking increase in the mRNA and protein levels of ANP and BNP was observed in *Fgf21*^-/-^ mice following TAC compared with those in the WT mice (Fig. 1, Fig. 1). Our data suggest that deleting FGF21 can worsen TAC-induced cardiac hypertrophy, thereby facilitating the onset of heart failure.

## Deletion of FGF21 results in mitophagy impairment and mitochondrial dysfunction in mice

**Fig. 2:**
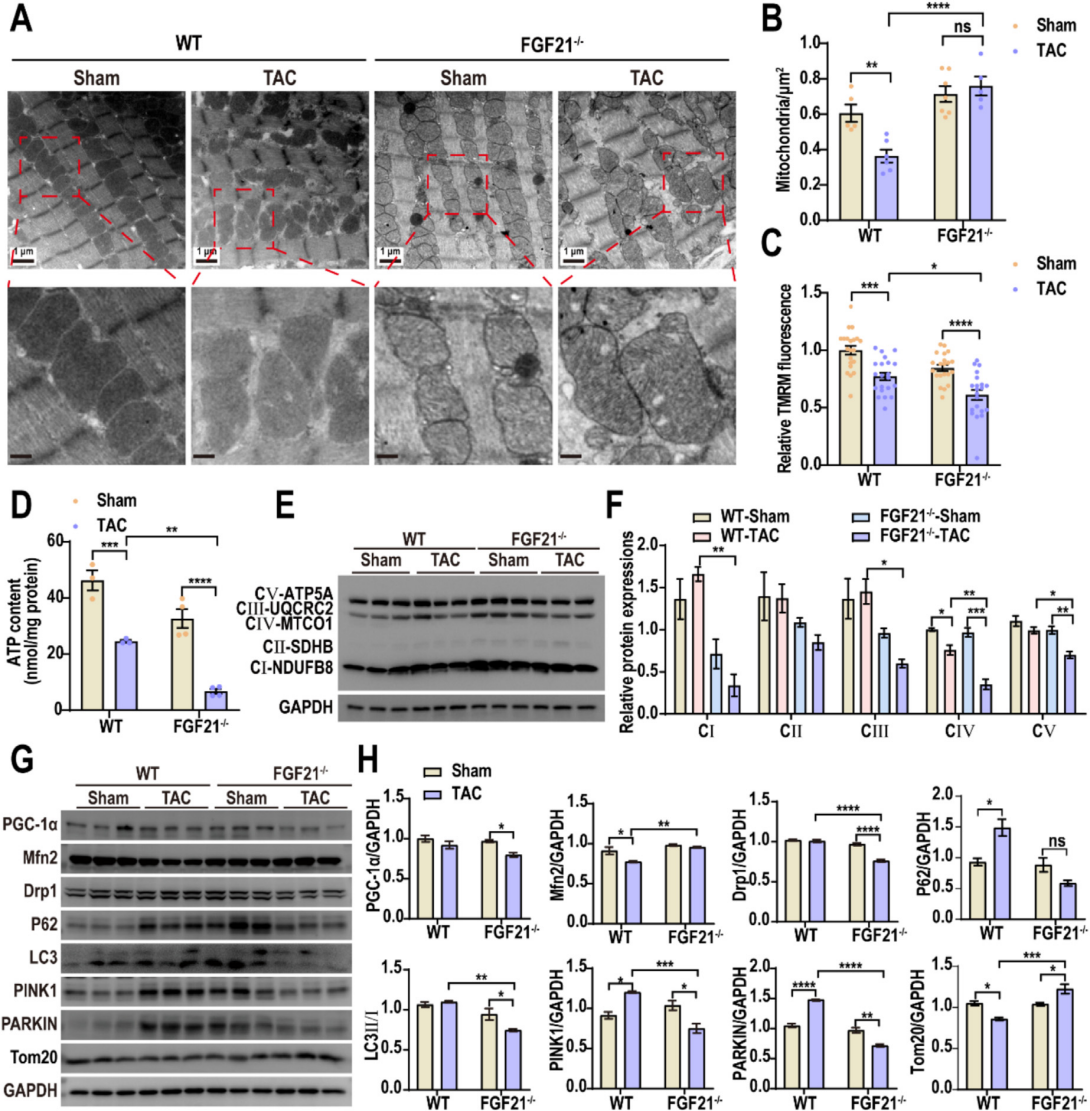
**Deletion of FGF21 inhibits mitophagy and impairs mitochondrial function in mice.** A. Representative images from TEM (n = 5–7), scale bar = 1 µm. B.Quantitative analysis of mitochondrial area for each group. C. Relative TMRM fluorescence in the indicated groups. D. ATP level in the indicated groups. E, F. Complex I, II, III, IV, and V protein levels in the indicated groups. Quantitative analysis was measured using ImageJ software. G, H. PGC-1α, Mfn2, Drp1, P62, LC3, PINK1, PARKIN, Tom20 protein levels in indicated groups. Quantitative analysis was measured using ImageJ software. All data represent mean ± SEM, and statistical significance was measured using the two-way ANOVA with the Bonferroni post-hoc test.

Mitochondria are the power factories of cardiomyocytes. Numerous studies have indicated that damaged and abnormal mitochondria frequently occur in cardiac hypertrophy and heart failure [30]. During the development of these conditions, both the quantity and structure of mitochondria change. Consequently, mitochondrial dysfunction can result in injury to cardiomyocytes, which further diminishes cardiac function [31]. Therefore, we performed transmission electron microscopy (TEM) to analyze changes in mitochondria in WT mice and *Fgf21^-/-^* mice following TAC. TEM revealed that the mitochondria in WT TAC mice were smaller than those in WT sham mice (Fig. 2, Fig. 2). However, the mitochondria in the *Fgf21^-/-^* TAC mice were larger than those in the *Fgf21^-/-^* sham mice (Fig. 2, Fig. 2). After TAC, the mitochondrial membrane potential was significantly lower in the *Fgf21^-/-^* mice than in the WT mice (Fig. 2C). These mitochondrial structural (disorganization, enlarged size) and functional (depolarization) impairments culminated in reduced mitochondrial function, which was further confirmed by the finding that ATP levels in *Fgf21^-/-^* mice following TAC were lower than those in WT mice (Fig. 2D). Moreover, we assessed the levels of mitochondrial respiratory electron transport chain (ETC) complexes, including those of complexes I, II, III, IV, and V. Western blotting revealed that complexes I, III, IV, and V were downregulated in the *Fgf21^-/-^* mice compared with WT mice post-TAC (Fig. 2, Fig. 2). Taken together, these findings suggest that compared with WT mice, mice lacking FGF21 after TAC exhibit dramatically impaired mitochondrial function.

MQC can maintain mitochondrial quantity and quality, which comprises mitochondrial biogenesis, fusion, fission, and mitophagy [32]. To investigate whether MQC is involved in mitochondrial impairment and dysfunction after overload pressure, we evaluated mitochondrial biogenesis, fusion, fission, and mitophagy levels in the TAC model. We found that the expression levels of PGC-1α and Drp1 were lower in *Fgf21^-/-^* mice than in WT mice following TAC (Fig. 2, Fig. 2). The expression of Mfn2 was downregulated in WT mice subjected to TAC. PINK1 and PARKIN levels were increased, and Tom20 expression was decreased in WT mice after TAC, compared to those in the sham group. Nevertheless, LC3, PINK1, and PARKIN were downregulated, and Tom20 was upregulated in *Fgf21^-/-^* mice post-TAC, compared with those in the sham group. These results show that mitochondrial biogenesis and dynamics are slightly inhibited in both WT and *Fgf21*^-/-^ mice following TAC, whereas mitophagy is enhanced in WT mice but considerably suppressed in *Fgf21^-/-^* mice. Collectively, our results identify impaired PINK1-dependent mitophagy as the key mechanism through which FGF21 depletion exacerbates TAC-induced heart failure.

## Knockdown of FGF21 impairs mitophagy and decreases pink1 levels in pe-induced NRCMs

**Fig. 3:**
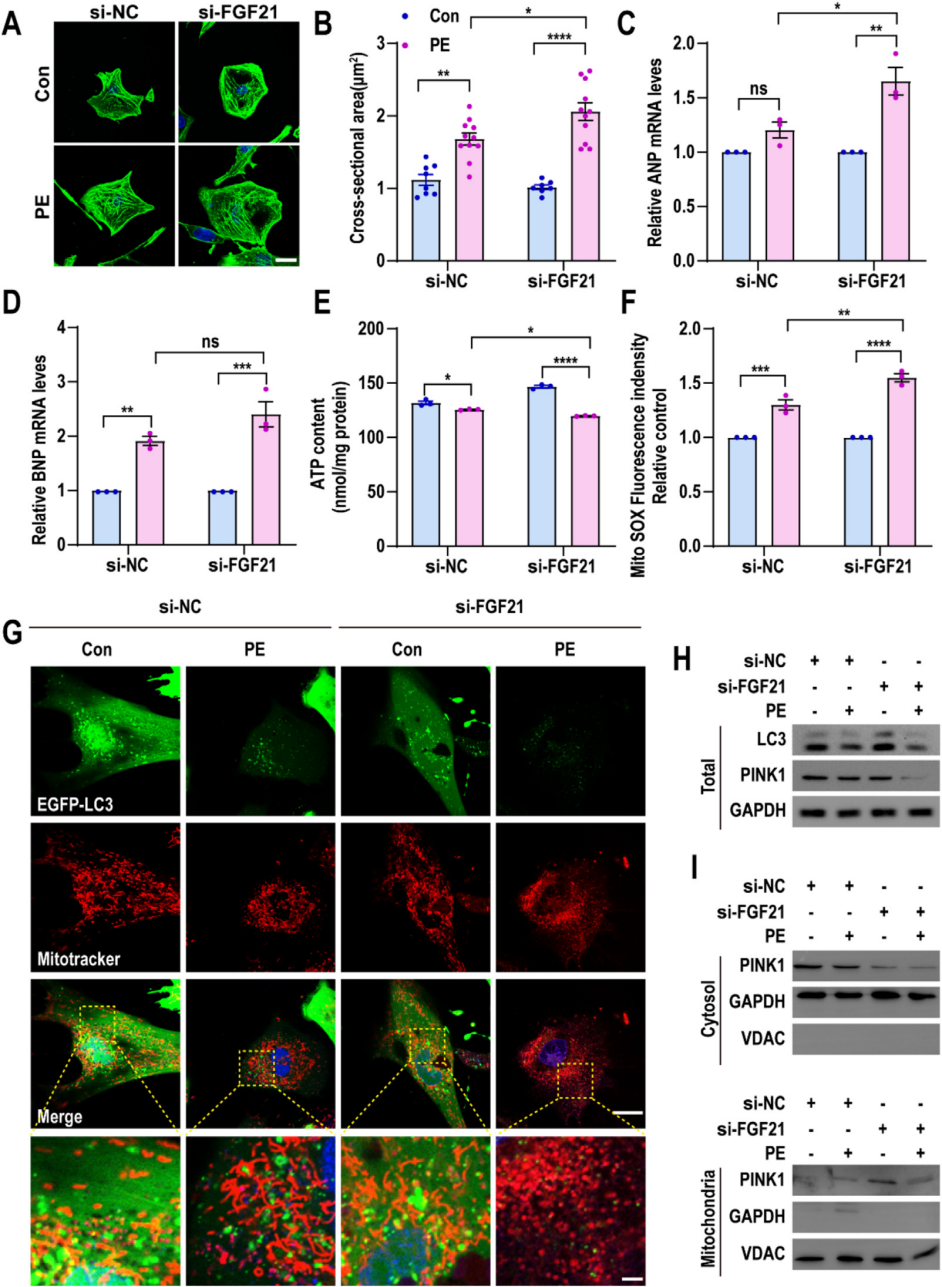
**Knockdown of FGF21 promotes cardiomyocyte hypertrophy and impairment of mitophagy and decreases PINK1 level in PE-induced NRCMs.** Neonatal rat cardiomyocytes were infected with si-NC or si-FGF21 and then treated with control or 100 μM PE for 24 h. A. Representative images of F-actin immunofluorescent. B. Quantitative analysis of cardiomyocyte cross area for each group, scale bar = 20 µm. C. Relative ANP mRNA level. D. Relative BNP mRNA level. E. ATP level in the indicated group. F. Fluorescence intensity of MitoSOX. G. si-NC or si-FGF21 were infected with adenovirus encoding LC3 for 24 h, then subjected to PE stimulation for 24 h. These cells were stained using MitoTracker Red dye, scale bar = 20 µm. H. LC3 and PINK1 protein levels. I. PINK1 protein levels in lysates of cytosolic and mitochondrial fractions. All data represent mean ± SEM, and statistical significance was measured using the two-way ANOVA with the Bonferroni post-test.

Next, we further evaluated the impact of FGF21 on the mitochondrial function of NRCMs. We treated NRCMs with the indicated concentration of PE. F-actin staining revealed that PE induced cardiomyocyte hypertrophy in a concentration-dependent manner (Fig. S2A, S2B). FGF21 levels were elevated by treatment with 100 μM PE (Fig. S2C, S2D). Therefore, 100 μM PE was used in subsequent experiments. Compared with the control treatment, PE treatment could increase the cross-sectional area of NRCMs, which was enhanced by the knockdown of FGF21 (Fig. 3, Fig. 3). Moreover, FGF21 deficiency increased PE-stimulated ANP and BNP mRNA expression (Fig. 3, Fig. 3). NRCMs were treated with PE to mimic similar changes, such as a reduction in ATP and an increase in ROS levels, in the context of cardiac hypertrophy in vivo. Therefore, we observed changes in ATP levels in PE-induced NRCMs. We found that the knockdown of FGF21 decreased ATP production in PE-induced NRCMs, indicating that the impairment of mitochondrial function was more severe in PE-induced si-FGF21 NRCMs than si-NC NRCMs (Fig. 3E). In addition, knockdown of FGF21 increased PE-induced total ROS levels (Fig. S2E) and mitochondria-derived ROS production (Fig. 3F). The production of large amounts of ROS, in turn, leads to damage to mitochondria. These data demonstrate that knockdown of FGF21 promotes PE-induced mitochondrial dysfunction in vitro.

Mitophagy impairment or defects contribute to mitochondrial dysfunction, leading to cellular dysfunction and ultimately, heart diseases [33]. In the mitophagy process, damaged mitochondria bind to LC3II puncta via specific autophagy-related receptors to form autophagosomes [34]. To confirm the effect of FGF21 on mitophagy in vitro, we measured the colocalization of EGFP-LC3-labeled autophagosomes and MitoTracker-labeled mitochondria. Immunofluorescence analysis revealed that the number of LC3 puncta colocalized with mitochondria was lower in FGF21-knockdown cells than in control cells after PE treatment, indicating that mitophagy was suppressed in cardiomyocytes (Fig. 3G). Finally, autophagosomes can fuse with lysosomes to degrade damaged mitochondria. Therefore, we observed the colocalization of LysoTracker-labeled lysosomes and MitoTracker-labeled mitochondria. FGF21 knockdown significantly impaired mitophagy, as evidenced by the reduced colocalization of lysosomes and mitochondria (Fig. S2F).

Additionally, we assessed changes in the molecular levels of mitophagy flux. The results revealed that the levels of LC3 and PINK1 were lower in the PE-induced NRCMs than in the si-NC NRCMs (Fig. 3H). As expected, the downregulation of FGF21 efficiently accelerated the abnormal expression of LC3II/LC3I and PINK1. Similarly, compared with that in the si-NC NRCMs, the PINK1 level in the mitochondrial fraction in the NRCMs infected with si-FGF21 was decreased after PE treatment (Fig. 3I). Together, these data demonstrate that the knockdown of FGF21 inhibits the PINK1-mediated mitophagy process under PE treatment.

## FGF21 rescues impaired mitophagy in NRCMs through the PINK1-mediated mitophagy pathway

**Fig. 4:**
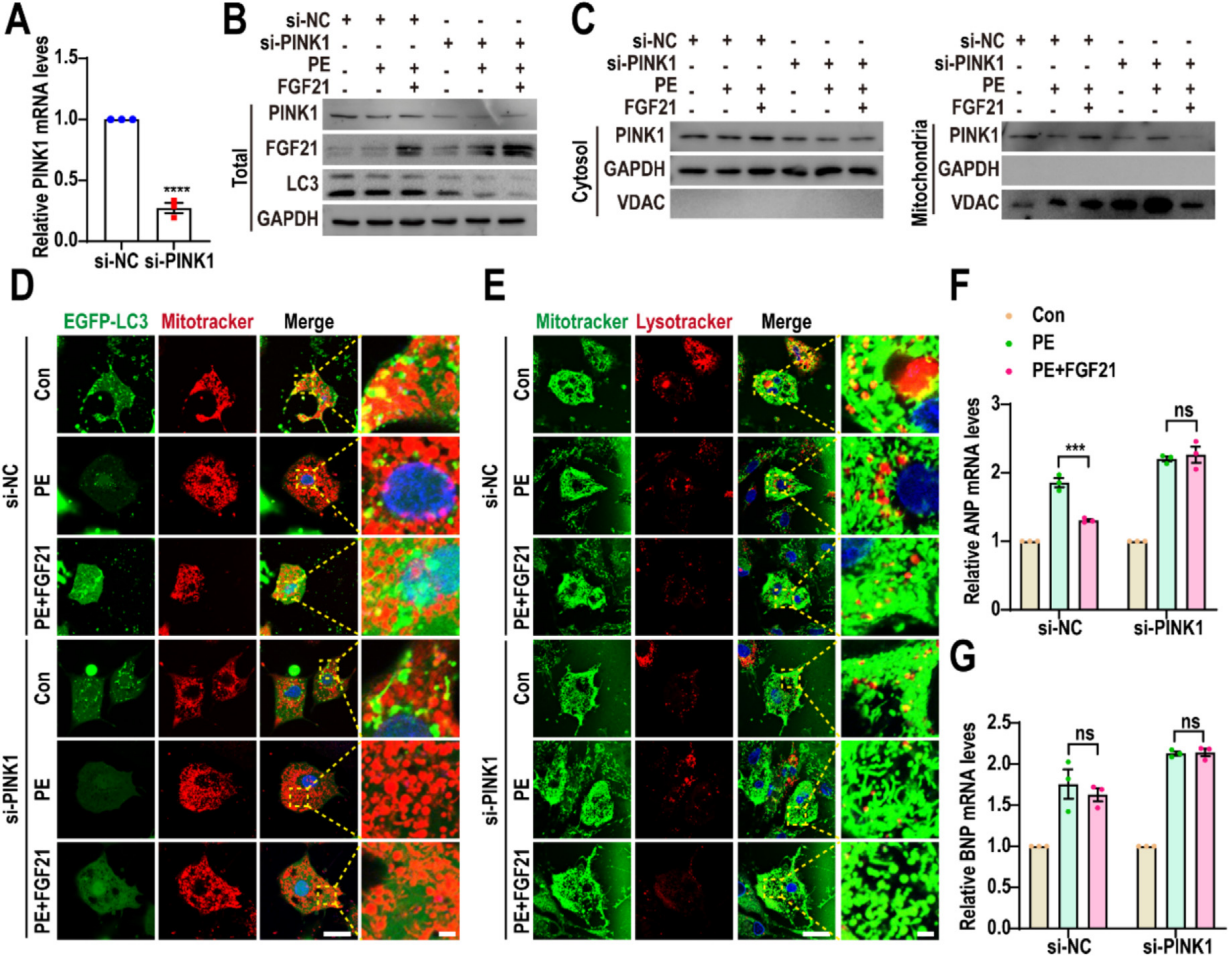
**Absence of PINK1 blocks FGF21-promoted mitophagy in PE-treated NRCMs.** Neonatal rat cardiomyocytes were infected with si-NC or si-PINK1, and then treated with control or 100 μM PE for 24 h, followed by vehicle or FGF21 protein. A. Relative PINK1 mRNA level. B. LC3 and PINK1, FGF21 protein levels. C. PINK1 protein level in lysates of cytosolic and mitochondrial fractions. D. si-NC and si-PINK1 NRCMs were infected with adenovirus encoding LC3 for 24 h, then subjected to 100 µM PE stimulation for 24 h, followed by vehicle or FGF21 protein. These cells were stained using MitoTracker Red dye, scale bar = 20 µm. E. Mitochondria and lysosomes were stained with MitoTracker green dye and LysoTracker Deep Red dye, scale bar = 20 µm. F. Relative ANP mRNA level. G. Relative BNP mRNA level. All data represent mean ± SEM, and statistical significance was measured using the two-way ANOVA with the Bonferroni post-test.

It has been reported that mitophagy can be divided into the PINK1/PARKIN-mediated pathway and the mediated pathway, including BNIP3/BNIP3L and FUNDC1 [35]. Our findings revealed that the levels of BNIP3L and FUNDC1 remained unaltered in *Fgf21^-/-^* mice compared with those in WT mice after TAC (Fig. S3A). In agreement with the in vivo results, there was no change in the levels of BNIP3L and FUNDC1 in NRCMs upon downregulation of FGF21 after PE treatment (Fig. S3B). Compared with those in WT mice, the levels of PINK1 and PARKIN in the *Fgf21^-/-^* mice significantly decreased after TAC. These findings suggest that FGF21 might regulate the PINK1-PARKIN pathway to mediate mitophagy under overload pressure. Therefore, we knocked down PINK1 via transfection with si-PINK1. PINK1 deficiency in cardiomyocytes was confirmed by qPCR (Fig. 4A). Next, we investigated whether PINK1 knockdown could abolish the regulatory effect of FGF21 on mitophagy. FGF21 administration markedly increased the ratio of LC3II/LC3Ⅰ and PINK1 in NRCMs treated with PE, but these increases were eliminated by knocking down PINK1 expression (Fig. 4B). Similarly, treatment of cardiomyocytes with FGF21 reversed the PE-induced downregulation of PINK1 expression in the mitochondrial fraction (Fig. 4C). We subsequently determined that more LC3 puncta colocalized with mitochondria upon FGF21 treatment in PE-induced cells, whereas this increase was blunted by a reduction in PINK1 expression (Fig. 4D). In agreement with these results, immunofluorescence analysis revealed that FGF21 treatment increased the colocalization of lysosomes and mitochondria in PE-treated cells, whereas the knockdown of PINK1 abolished the effect of FGF21 (Fig. 4E). We also found that PINK1 knockdown significantly inhibited the FGF21-mediated decrease in ANP and BNP expression in PE-treated cells (Fig. 4, Fig. 4), which indicates that FGF21 can reverse PE-mediated cardiomyocyte hypertrophy by modulating PINK1-induced mitophagy.

## Protective effects of FGF21 against cardiac hypertrophy are abolished in the absence of PINK1 in mice

**Fig. 5:**
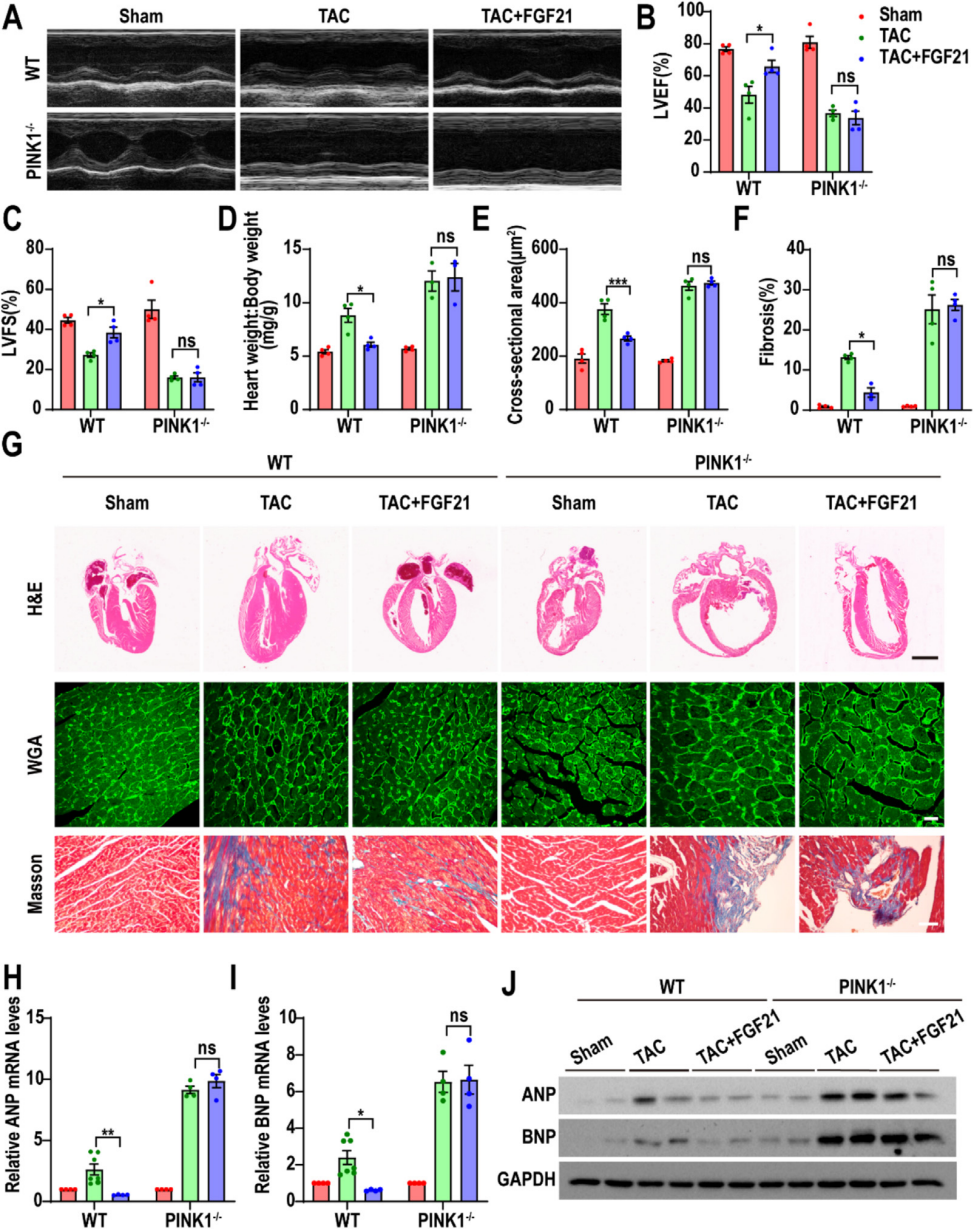
**The protective effects of FGF21 against TAC-induced cardiac hypertrophy are abolished in the absence of PINK1 in mice.** Ten-week-old WT and *Pink1^-/-^* mice underwent sham or TAC surgery. One week after the TAC, some TAC groups were subcutaneously injected with saline, and other TAC groups were subcutaneously injected with Fc-FGF21 fusion protein (Efruxifermin). The injection is once a week for four weeks. A. Representative M−mode echocardiography of the left ventricle of WT or *Pink1^-/-^* mice in the indicated groups. B. Measurement of LVEF in the indicated groups. C. Measurement of LVFS in the indicated groups. D. Ratio of HW to BW. E. Quantitative analysis of cardiomyocyte cross-sectional area for each group (n = 3, 4). F. Quantitative analysis of interstitial fibrosis for each group. G. Representative image of HE staining, scale bar = 2 mm, and WGA immunofluorescent staining, scale bar = 20 µm, and Masson's Trichrome staining, scale bar 100 µm (n = 4). H, I. ANP and BNP mRNA levels in the indicated group. J. ANP and BNP protein levels in the indicated group. All data represent mean ± SEM, and statistical significance was measured using the two-way ANOVA with the Bonferroni post-hoc test.

Further exploration of the mechanism by which FGF21 protects against cardiac hypertrophy via PINK1 regulation in mice is needed. WT mice or *Pink1^-/-^* mice underwent TAC. TAC-induced WT or *Pink1^-/-^* mice were randomly treated with the Fc-FGF21 fusion protein or saline once a week for four weeks (Fig. S4A). Echocardiographic analysis revealed decreases in the LVEF and LVFS in WT mice after TAC (Fig. 5A–C). Compared with saline treatment, FGF21 treatment reduced the HW/BW ratio, cardiomyocyte size, and degree of fibrosis in TAC-induced WT mice (Fig. 5D–G). Additionally, ANP and BNP mRNA and protein levels were decreased (Fig. 5H–J) in TAC-induced WT mice treated with FGF21. Each of these FGF21-mediated effects was abolished by the deletion of PINK1. To confirm whether FGF21 in TAC-induced WT mice could improve mitochondrial mitophagy, we evaluated mitochondrial autophagic vacuoles and ETC complex subunits. Compared with saline treatment, FGF21 treatment increased the incorporation of mitochondria into autophagic vacuoles (Fig. S4B, S4C) and mitochondrial respiratory electron transport chain complex protein levels (Fig. S4D), whereas these effects were blunted by the knockout of PINK1. We further detected the expression levels of crucial genes of OXPHOS subunits, electron carriers, mtDNA, the TCA cycle, metabolism, and Fe-S. The results revealed that the expression of these genes decreased in TAC-induced WT mice compared to the saline-treated mice, and that FGF21 treatment reversed these changes (Fig. S4E). Compared with WT mice, *Pink1^-/-^* mice displayed a significant reduction in expression after TAC. FGF21 administration did not counteract this outcome (Fig. S4E). Taken together, these findings suggest that the depletion of PINK1 abolishes FGF21-mediated mitophagy in vivo, thereby accelerating the progression of myocardial hypertrophy.

## Activation of PINK1-mediated mitophagy prevents from TAC-induced heart failure in *Fgf21*^-/-^ mice

**Fig. 6:**
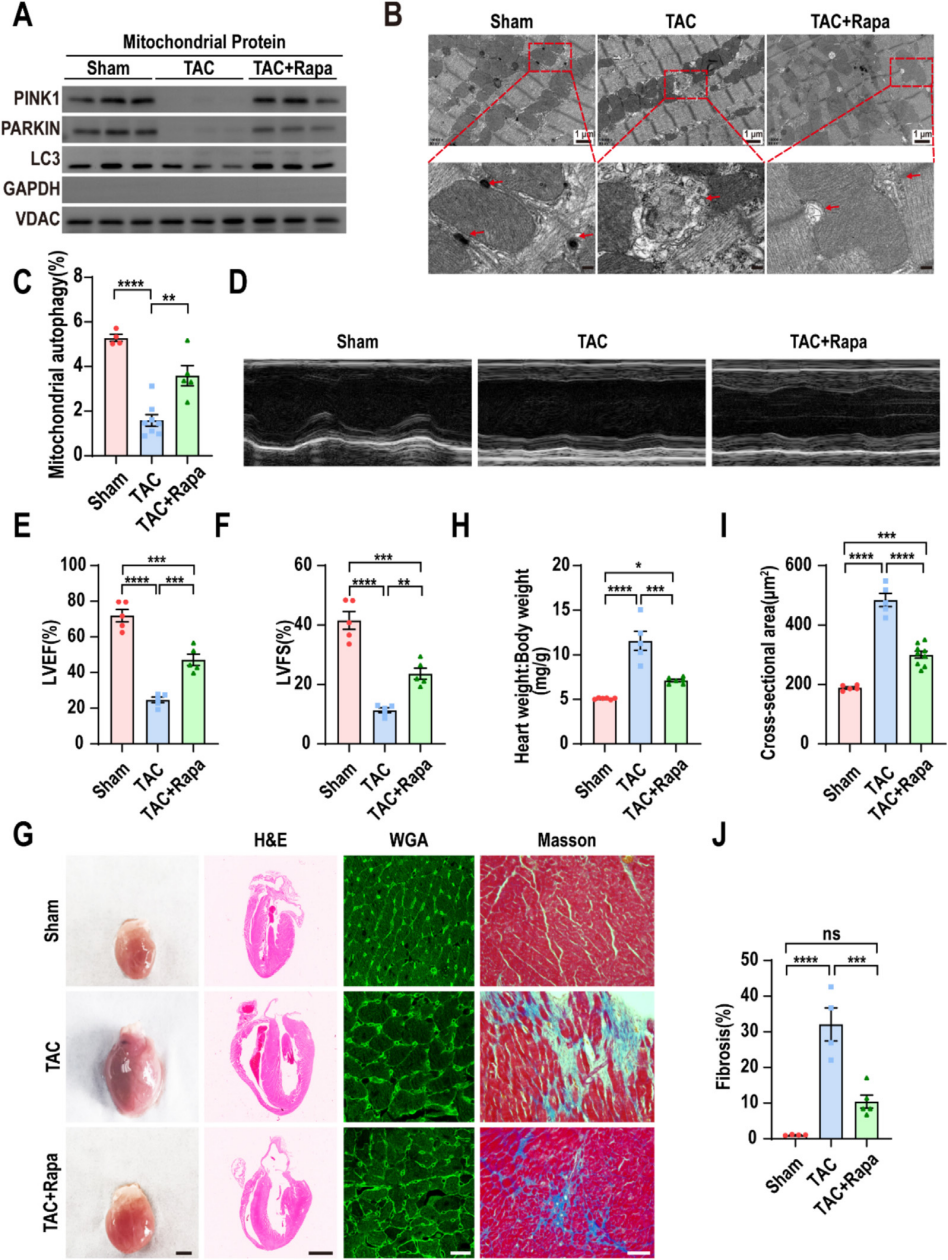
**The activation of PINK1-mediated mitophagy prevents *Fgf21^-/-^* mice from TAC-induced heart failure.** Ten-week-old *Fgf21^-/-^* mice underwent sham or TAC surgery. Three weeks after the TAC, the sham and TAC groups were intraperitoneally injected with solvent, and the other TAC group was intraperitoneally injected with rapamycin for two weeks. A. PINK1 and PARKIN, LC3 protein levels in lysates of mitochondrial fractions. B. Representative images from TEM (n = 3,7), scale bar = 1 µm. C. Quantitative analysis of the number of autophagosomes containing mitochondria for each group. D. Representative M−mode echocardiography of the left ventricle of *Fgf21^-/-^* mice after sham or TAC surgery or TAC surgery treated with rapamycin. E. Measurement of LVEF in the indicated groups. F. Measurement of LVFS in the indicated groups. G. Gross hearts, scale bar = 2 mm and representative image of HE staining, scale bar = 2 mm (n = 5, 6), representative image of WGA immunofluorescent staining (n = 5, 9), scale bar = 20 µm and representative images of Masson's Trichrome staining (n = 4, 5), scale bar = 100 µm. H. Ratio of HW to BW. I. Quantitative analysis of cardiomyocyte cross-sectional area for each group. J. Quantitative analysis of interstitial fibrosis for each group. All data represent mean ± SEM, and statistical significance was measured using the One-way ANOVA followed by Dunnett's post-hoc test.

Accumulating studies have shown that Rapa can stimulate mitophagy [36,37]. Furthermore, Rapa promotes mitophagy by increasing PINK1 and PARKIN levels. Therefore, we first tested whether PINK1 expression was elevated by Rapa and whether PINK1-mediated mitophagy was enhanced [38]. The findings revealed that Rapa increased PINK1 mRNA and protein levels (Fig. S5A, S5B). In addition, immunofluorescence staining revealed that Rapa increased the PINK1 level and promoted the transfer of PINK1 to mitochondria (Fig. S5C). In vitro, mitophagy was decreased in si-FGF21 NRCMs treated with PE, whereas Rapa reversed this change (Fig. S5D). In vivo, *Fgf21^-/-^* mice were treated with Rapa via intraperitoneal injection three weeks after TAC. Compared with those in the TAC group, the LC3, Beclin1, PINK1, and PARKIN levels increased, while the P62 level in the *Fgf21^-/-^* group treated with Rapa decreased (Fig. S5E). Moreover, compared with those in the TAC group, the levels of PINK1, PARKIN, and LC3 substantially increased in the mitochondrial fractions of FGF21 knockout mice treated with Rapa following TAC (Fig. 6A). Mitochondrial incorporation into autophagic vacuoles was significantly greater in mice lacking FGF21 treated with Rapa after TAC than in mice lacking FGF21 induced by TAC (Fig. 6, Fig. 6). These findings suggest that Rapa stimulates PINK1-mediated mitophagy. We next investigated whether the increase in mitophagy rescued mitochondrial dysfunction and cardiac function damage in FGF21 knockout mice under overload pressure. The reduction in ATP production was reversed in *Fgf21^-/-^* mice post-TAC after Rapa injection, suggesting that Rapa improved mitochondrial function (Fig. S5F, S5G). Furthermore, we investigated the change in cardiac function. The LVEF and LVFS decreased dramatically in *Fgf21^-/-^* mice after TAC, but Rapa injection restored cardiac function (Fig. 6, Fig. 6). We also observed that *Fgf21^-/-^* mice treated with Rapa were protected against TAC-induced heart failure as evidenced by decreased HW/BW (Fig. 6, Fig. 6), decreased cardiomyocyte size and fibrosis levels (Fig. 6, Fig. 6, Fig. 6), and reduced ANP and BNP levels (Fig. S5H–J). Rapa activated PINK1-mediated mitophagy, but it did not directly target mitochondria. We used PMI, a P62-targeted mitophagy inducer, to enhance mitophagy. FGF21-deficient mice were treated with PMI via intraperitoneal injection one week after TAC surgery. The levels of Beclin1, PINK1, and PARKIN increased, whereas the levels of P62 and Tom20 decreased in the *Fgf21^-/-^* mice treated with PMI after TAC compared with those in the *Fgf21^-/-^* mice after TAC (Fig. S6A). Compared with those in TAC-induced *Fgf21^-/-^* mice, the protein levels of the ETC complex increased in *Fgf21*^-/-^ mice treated with PMI after TAC (Fig. S6B). These data demonstrate that mitophagy and mitochondrial function are rescued by PMI treatment. Moreover, cardiac hypertrophy improved in the PMI-mediated mice after TAC, as evidenced by a reduction in cardiac volume and cardiomyocyte size (Fig. S6C, S6D). Collectively, these findings demonstrate that FGF21 deficiency leads to impaired mitophagy and cardiac function after TAC, both of which can be rescued by restoring mitophagy through Rapa or PMI treatment.

## FGFR1 is essential for the mitophagy-promoting effect of FGF21 in cardiac myocytes

**Fig. 7:**
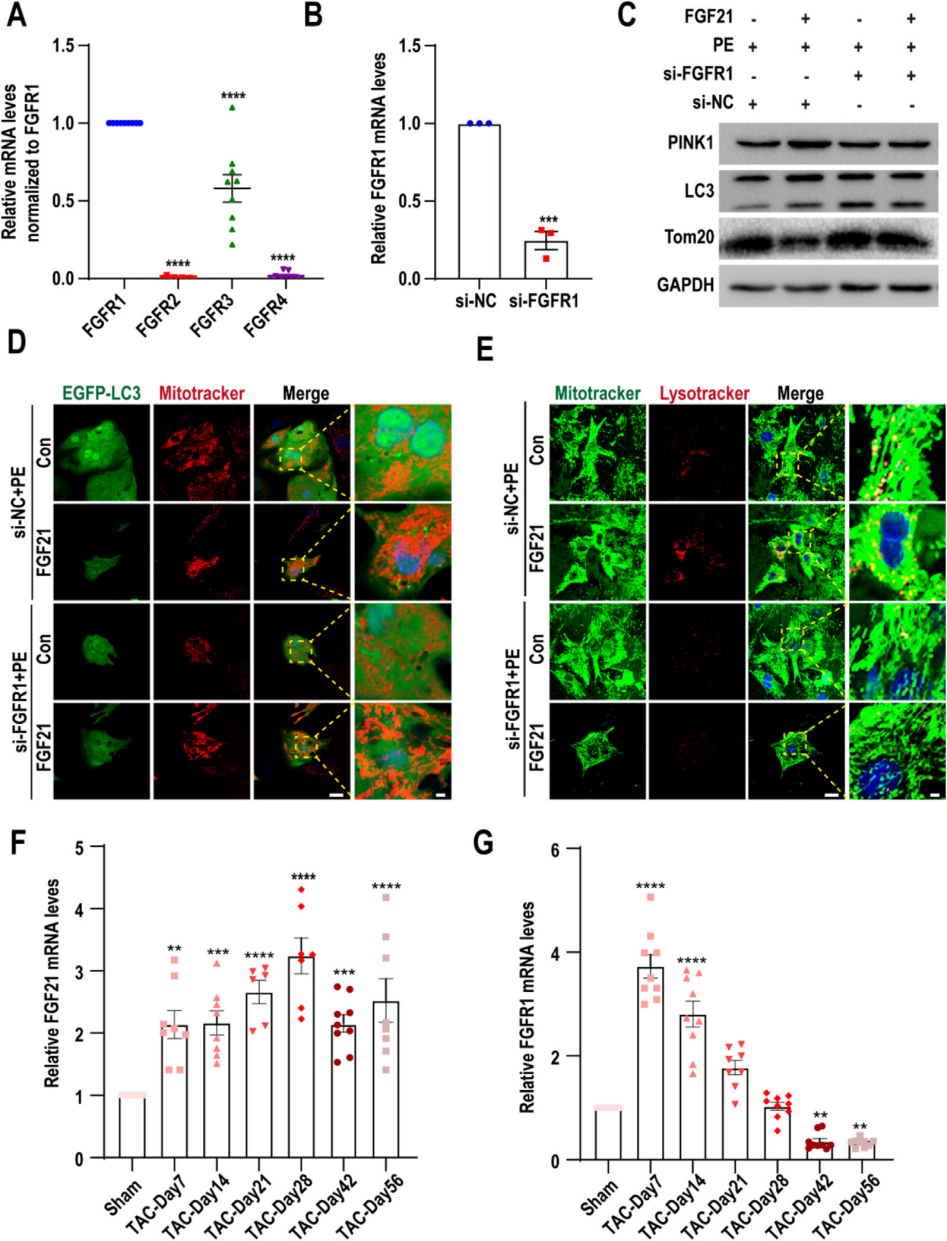
**FGFR1 is necessary for FGF21-mediated mitophagy.** A. FGFR1, FGFR2, FGFR3, and FGFR4 mRNA levels in the heart of WT mice. B. Neonatal rat cardiomyocytes were infected with si-NC or si-FGFR1. Relative FGFR1 mRNA level. C. Neonatal rat cardiomyocytes were infected with si-NC or si-FGFR1, and then treated with 100 μM PE for 24 h, followed by vehicle or FGF21 protein, LC3, PINK1, and Tom20 protein levels. D. si-NC and si-FGFR1 NRCMs were infected with adenovirus encoding LC3 for 24 h, then subjected to 100 μM PE stimulation for 24 h, followed by vehicle or FGF21 protein. These cells were stained using MitoTracker Red dye, scale bar = 20 µm. E. Mitochondria and lysosomes were stained with MitoTracker green dye and LysoTracker Deep Red dye, scale bar = 20 µm. F. Relative FGF21 mRNA level after TAC at different times. G. Relative FGFR1 mRNA level after TAC at different times. All data represent mean ± SEM, and statistical significance was measured using the One-way ANOVA followed by Dunnett's post-hoc test and the two-way ANOVA with the Bonferroni post-test.

FGFR is necessary for the ability and sensitivity of FGF21 to exert its effects. FGF21 stimulates different FGFRs under different conditions. For example, FGF21 induces FGFR4 to promote diabetic cardiomyopathy [39]. In addition, FGF21 activated FGFR1 in NRCMs under basal conditions [17]. FGF21 is strongly positively related to FGFR3 in heart failure patients [40]. Studies have shown that FGFR1, FGFR3, and FGFR4 are significantly expressed in cardiac myocytes, whereas FGFR2 is barely expressed [41,42]. Similarly, we detected FGFR1 and FGFR3 expression in cardiac tissue, with FGFR1 expression being higher than that of FGFR3 (Fig. 7A). To explore whether FGFR1 or FGFR3 affects cardiac hypertrophy and heart failure, we knocked down FGFR1 or FGFR3 by siRNA transfection (Fig. 7B, Fig. S7A). Only the knockdown of FGFR1 inhibited the increase in LC3Ⅱ/Ⅰ and PINK1 levels and decrease in Tom20 levels induced by FGF21 in PE-mediated NRCMs (Fig. 7C, Fig. S7B). Immunofluorescence analysis revealed that FGF21 treatment increased the colocalization of LC3 and mitochondria and the colocalization of lysosomes and mitochondria in PE-treated NRCMs, whereas FGFR1 deficiency abolished the effect of FGF21 (Fig. 7, Fig. 7). The quantity of lysosomes was significantly decreased. To assess lysosomal status, we detected the expression levels of lysosome-related marker genes (LAMP1). The results showed that FGF21 treatment significantly upregulated the expression of LAMP1, suggesting that it could effectively promote lysosome biogenesis or functional recovery (Fig. S7C). However, FGFR1 knockdown completely abolished the upregulatory effect of FGF21 on LAMP1, and simultaneously blocked its promotional effect on lysosomes. Taken together, these findings suggest that FGF21-promoted mitophagy is inhibited in NRCMs when FGFR1 is absent.

The antihypertrophic effects of FGF21 have been demonstrated previously [43,44]. In contrast, FGF21 levels were paradoxically increased in HF, indicating an FGF21-resistant state [45,46]. We hypothesized that FGFR1 deficiency abolished the protective effect of FGF21 in heart failure. Therefore, we first detected FGF21 levels during the progression of cardiac hypertrophy and heart failure. As expected, FGF21 levels were elevated in TAC-induced mice at different time points (7 days, 14 days, 21 days, 28 days, 42 days, and 56 days) (Fig. 7F). Next, we found that the expression of FGFR1 significantly increased at 7 days post-TAC but was markedly decreased by 42 days post-TAC (Fig. 7G). FGFR3 expression transiently increased at 7 days post-TAC, returned to baseline by 14 days, and significantly decreased by 42 days (Fig. S7D). In addition, we analyzed the characteristics of differentially expressed genes (DEGs) in HF patients using the Gene Expression Omnibus (GEO) database. Specifically, in the validation dataset GSE120895, the expression of FGF21 in HF patients was significantly increased (Fig. S7E); conversely, the expression of FGFR1 was decreased in another validation dataset GSE5406 (Fig. S7F). These clinical transcriptomic data are consistent with the results obtained from our animal model experiments. These findings demonstrate that the expression of FGFR1 and FGF21 exhibits opposite trends during HF, providing strong evidence for the role of the FGF21-resistant state.

## Discussion

Despite intensive studies on the metabolic mechanism of FGF21, understanding of its molecular mechanism in the pathogenesis of cardiac hypertrophy and heart failure remains relatively limited. In this study, FGF21 deficiency markedly exacerbated cardiac hypertrophy and increased cardiac damage in TAC-induced mice via the inhibition of PINK1 levels. Treatment with FGF21 significantly improved these negative effects, suggesting that FGF21 is a physiological protector against cardiac hypertrophy. The protective role of FGF21 in cardiac hypertrophy is consistent with that reported by the Planavila group [17]. The expression of FGF21 increases in cardiac hypertrophy, indicating a compensatory response under these conditions. In support of this view, upregulation of FGF21 serves as a compensatory mechanism to protect against hypertensive heart disease [47,48], diabetic cardiomyopathy [49], and metabolic dysfunction-associated steatohepatitis [50].

Accumulating evidence highlights that mitochondrial dysfunction serves as a key driver in the progression from cardiac hypertrophy to heart failure, underscoring its central role in the transition of myocardial remodeling to overt impaired cardiac function [51]. With decreasing mitochondrial volume density, mitochondrial cristae are damaged, and shape alternates during hypertrophy and heart failure, accompanied by reduced ATP production, diminished activity of myocardial mitochondrial enzyme complexes, and restrained oxidative phosphorylation. In the current study, our results revealed obvious mitochondrial abnormalities, in terms of structure and function, in *Ffg21^−^*^/^*^−^* mice post-TAC compared with WT mice (Fig. 2). The TEM results revealed that the number of elongated mitochondria increased in the *Fgf21^-/-^* mice post-TAC, although these changes did not reach statistical significance (Fig. 2A). Notably, this elongated mitochondrial morphology is a phenotypic feature typically linked to preserved expression of Mfn2 and reduced levels of Drp1, which we observed in *Fgf21^−^*^/^*^−^* mice (Fig. 2G). This association between FGF21 deficiency and altered mitochondrial fusion–fission balance is further supported by a previous study, which demonstrated that depletion of FGF21 induces mitochondrial fusion in mesenchymal stem cells [52]. In contrast, WT mice post-TAC displayed a significant accumulation of small, fragmented mitochondria—an alteration tightly linked to the decrease in Mfn2 levels (Fig. 2G). These data indicate that FGF21 influences mitochondrial structural integrity and subsequent functional outcomes.

Mitophagy selectively removes damaged mitochondria, which maintains mitochondrial homeostasis. Previous studies have demonstrated that PINK1 expression is significantly downregulated during the progression of heart failure, and that its activity is essential for cardiac remodeling [53]. In our research, we observed that TAC promoted the activity of the PINK1/PARKIN pathway in WT mice but suppressed it in *Fgf21^-/-^* mice (Fig. 2G). Notably, FGF21 treatment effectively reversed TAC-induced cardiac function damage and mitophagy suppression; however, these rescue effects were completely abrogated when PINK1 was depleted (Fig. 5). These findings confirm that FGF21 modulates mitophagy in a PINK1-dependent manner. Mechanistically, this regulatory effect of FGF21 can be linked to the AMP-activated protein kinase (AMPK) pathway, as supported by existing studies: the Wang group reported that AMPKα2 directly activates PINK1, which in turn recruits PARKIN to mitochondria, enhances mitophagy, and ultimately inhibits heart failure progression [54]. This AMPK-PINK1 axis is not limited to cardiac contexts. In other disease models, AMPK activation has been shown to regulate the expression level and activity of PINK1, thereby promoting mitophagy [55,56]. Importantly, accumulating evidence has established that FGF21 exerts its biological functions primarily by activating the AMPK pathway [[57], [58], [59], [60]]. Together, these observations reveal a coherent regulatory cascade in which FGF21 activates AMPK, which in turn modulates PINK1-mediated mitophagy to protect cardiac function under pressure overload.

Biologically, FGF21 relies on FGFR to activate downstream pathways. The Planavila group reported that FGF21 treatment activated FGFR1 in NRCMs [17]. In further support of the role of FGFR1in FGF21-mediated cardiac effects, FGF21 exerts potent cardioprotective effects in type 2 diabetes mice through the FGFR1-AMPK pathway [61]. Our own experimental data further reinforce this critical role of FGFR1: FGFR1 knockdown completely abrogated the FGF21-induced activation of mitophagy. Together, these findings collectively suggest that the FGF21-FGFR1-AMPK-PINK1 axis may represent a key signaling pathway in cardiac hypertrophy. However, there is a strikingly paradoxical phenomenon. While FGF21 levels were consistently elevated during the progression of HF, FGFR1 levels were significantly reduced at 42 days post-TAC (Fig. 7, Fig. 7). This paradox is also reflected in clinical results, that is, an increase in circulating FGF21 levels and cardiac FGF21 mRNA expression in HF patients [[62], [63], [64]], yet elevated FGF21 is associated with poor clinical outcomes in these patients. This phenomenon has also been observed in other disease contexts, such as diabetes and its cardiovascular complications [65,66]. Based on our experimental results, we hypothesize that the downregulation of FGFR1 may be a key driver of FGF21 resistance. This hypothesis provides a mechanistic explanation for the paradox of elevated FGF21 failing to exert protective effects in cardiac hypertrophy and HF. In this study, the mechanism underlying the downregulation of FGFR1 remains unclear. Previous studies have suggested that in myocardial tissue, FGFR1 expression may be reduced through epigenetic mechanisms, such as DNA methylation [67] or histone deacetylation [68]. Additionally, in hepatocytes and embryonic neural stem cells, neuronal precursor cell-expressed developmentally downregulated 4 (NEDD4) directly binds FGFR1 and promotes its ubiquitin-proteasomal degradation [69,70]. Notably, a recent study reported a significant increase in NEDD4 expression four weeks after TAC [71]. These findings suggest that the reduced FGFR1 level may result from NEDD4-mediated ubiquitination in TAC-induced heart failure. Furthermore, FGFR1 expression is also regulated by microRNAs such as miR-497 and miR-133 [72,73]. Collectively, these data provide a theoretical basis for future investigations into the mechanisms responsible for FGFR1 downregulation during the progression of heart failure.

## Limitations

First, the biological sample size in some of our experiments, such as those used for TEM analysis and histopathological staining, was relatively small. Therefore, future studies will require larger sample cohorts to further increase the statistical robustness. Second, we analyzed only the expression patterns of FGF21 and FGFR1 in HF patients using publicly available data from the GEO database, and did not generate data from our own independent clinical trials. Given these limitations, it will be necessary to conduct prospective clinical trials in the future. These trials should involve a well-characterized HF patient cohort to validate the observed FGF21/FGFR1 expression trends and clarify their clinical relevance to HF progression or prognosis. Third, we utilized an in vivo TAC-induced mouse model and an in vitro PE-induced cardiomyocyte model in this study. The in vivo model reflects pathological regulatory responses, whereas the in vitro model focuses on isolated cardiomyocytes under controlled stress conditions. Notably, in vitro experimental results are available only for the FGF21-FGFR1 signaling axis; additional validation using FGFR1 conditional knockout mouse models is still needed to confirm whether FGFR1 downregulation directly mediates FGF21 resistance in vivo. Finally, the molecular mechanism underlying FGFR1 downregulation in HF remains elusive. For instance, it is unclear whether this downregulation arises from epigenetic silencing, post-transcriptional regulation, or accelerated protein degradation. This gap warrants further mechanistic investigation to comprehensively elucidate the regulatory network governing FGFR1 expression in HF.

## Compliance with ethics requirements

This article does not contain any studies with human or animal subjects.

## Declaration of competing interest

The authors declare that they have no known competing financial interests or personal relationships that could have appeared to influence the work reported in this paper.
