# Supplementary material for: FGF21-FGFR1 signaling protects against cardiac hypertrophy by regulating PINK1-mediated mitophagy pathway
Source: J Adv Res. 2025 Oct 29;85:1009–23. doi: 10.1016/j.jare.2025.10.053 (PMC13316545; doi:10.1016/j.jare.2025.10.053)
Supplement: Supplementary Data 1 [file mmc1.docx]

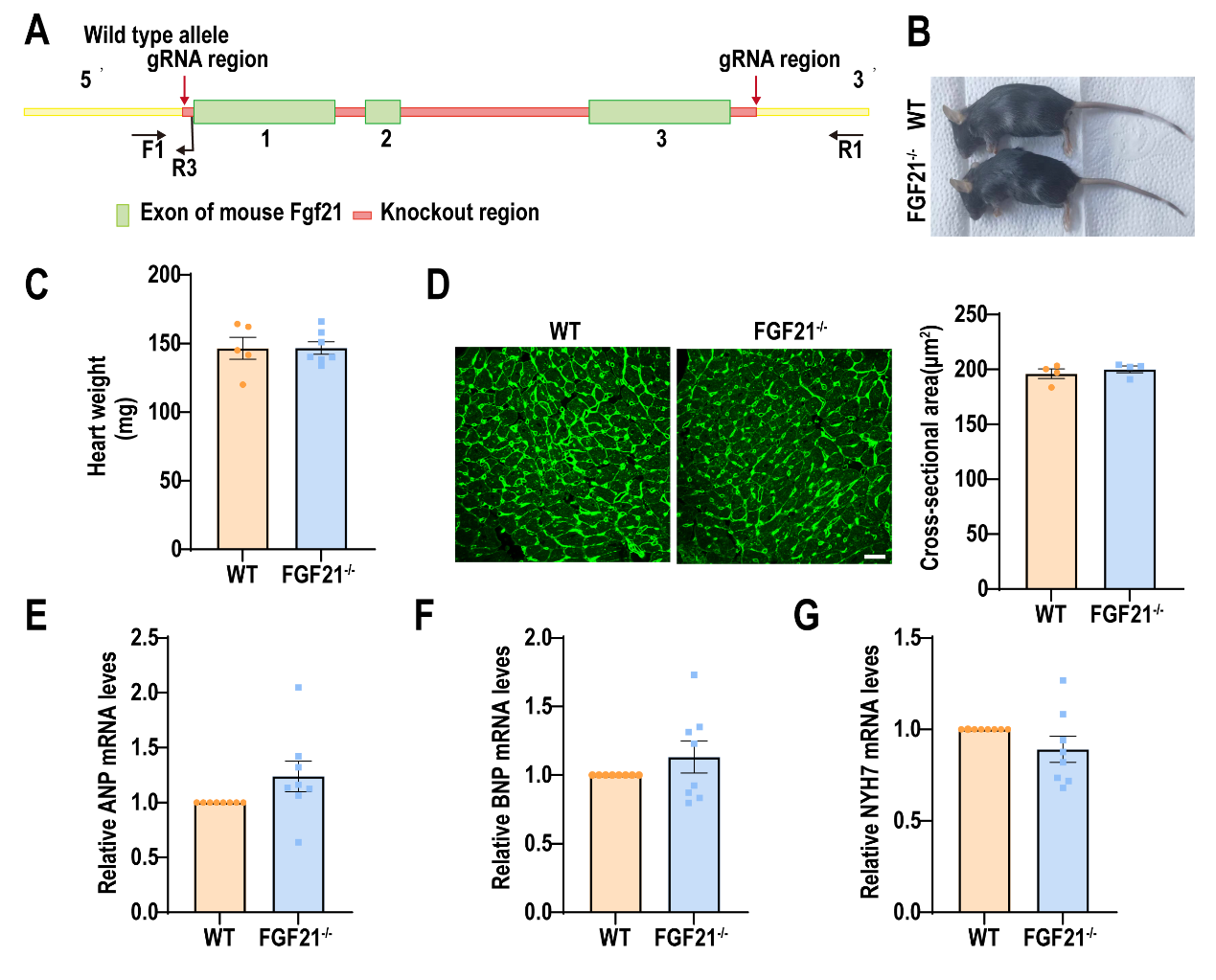


**Figure S1: The deletion of FGF21 does not change body shape, heart weight and volume, and expression of heart failure markers in ten-week-old *Fgf21^-/-^* mice.** A. *Fgf21^-/-^* mice genotyping strategy. B. Body shape of WT and *Fgf21^-/-^*. C. The heart weight of WT and *Fgf21^-/-^* mice (n = 5, 13). D. Representative image of WGA immunofluorescent staining (n = 4), scale bar = 20 µm, and quantitative analysis of cardiomyocyte cross-sectional area for each group. E–G. The mRNA expressions of hypertrophic markers (ANP, BNP, MYH7) were detected by qPCR. All data represent mean ± SEM, and statistical significance was measured using the One-way ANOVA followed by Dunnett’s post-hoc test.


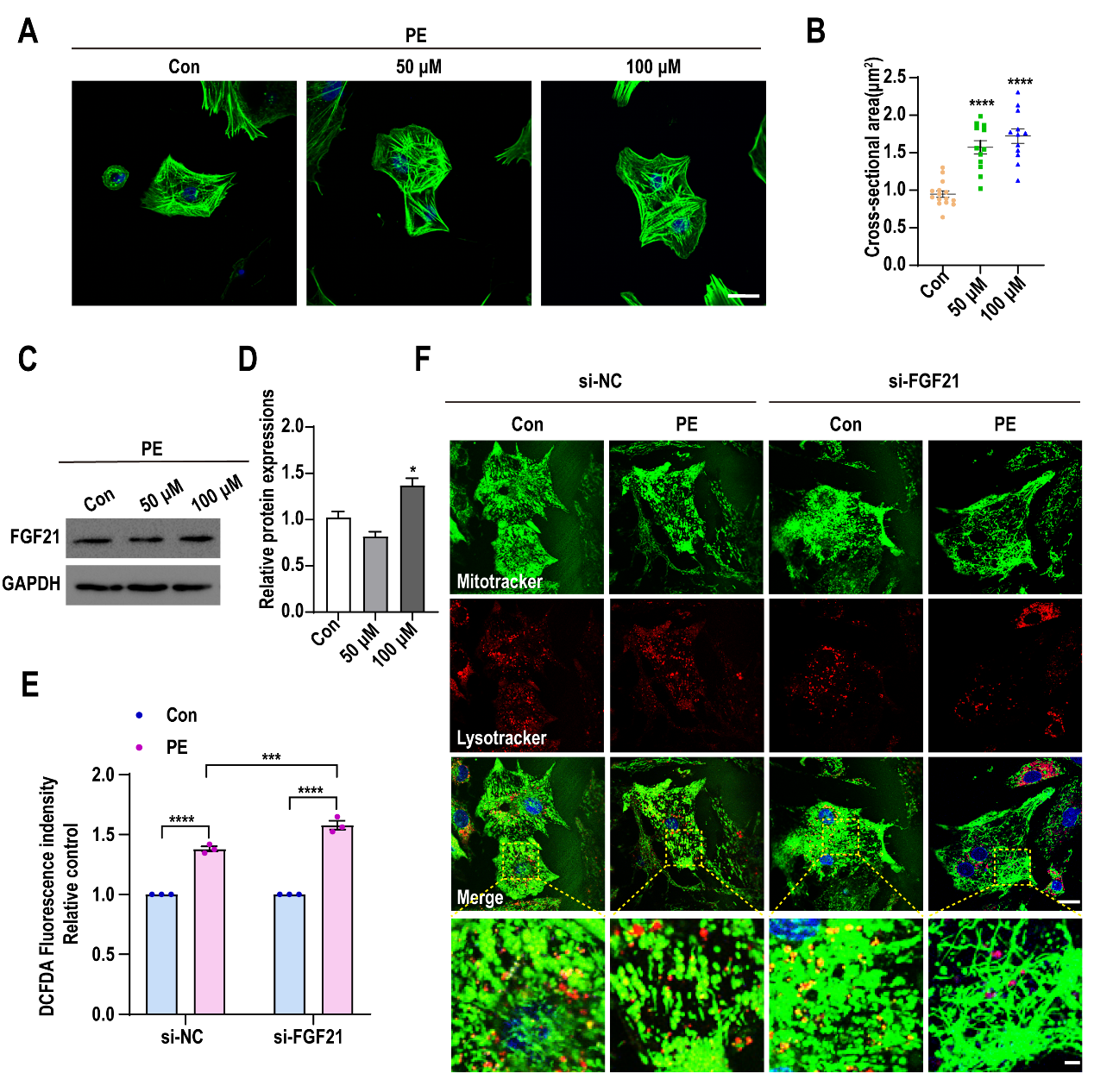


**Figure S2: PE induces cardiomyocyte enlargement in a concentration-dependent manner.** A**.** Neonatal rat cardiomyocytes were treated with PE in the indicated concentrations. Representative images of F-actin immunofluorescent, scale bar = 20 µm. B. Quantitative analysis of cardiomyocyte cross area for each group. C. The protein levels of FGF21. D. Quantitative analysis of FGF21 was measured by ImageJ software. E. Fluorescence intensity of DCFDA. F. Mitochondria and lysosomes were stained with MitoTracker green dye and LysoTracker Deep Red dye, scale bar = 20 μm. All data represent mean ± SEM, and statistical significance was measured using the One-way ANOVA followed by Dunnett’s post-hoc test or the two-way ANOVA with the Bonferroni post-hoc test.


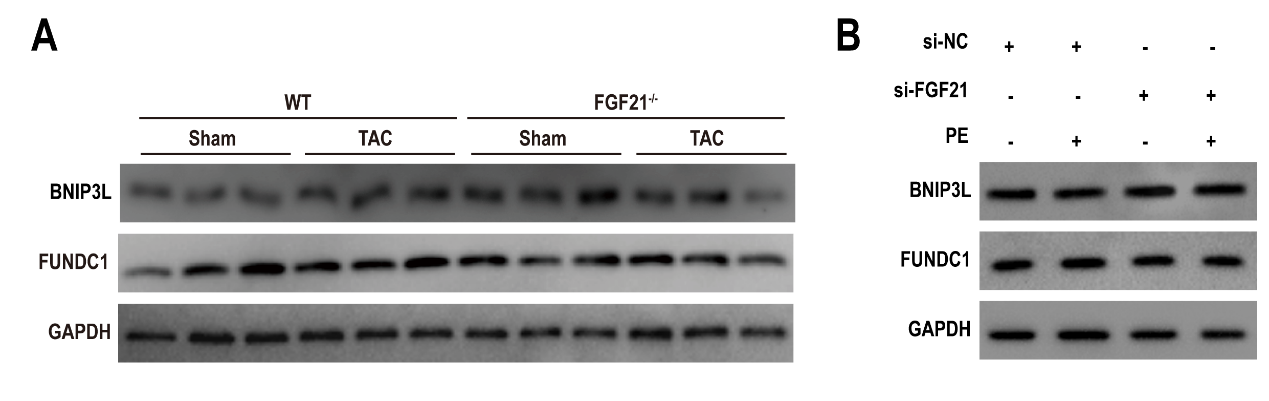


**Figure S3: The decrease of FGF21 does not affect the expression of BNIP3L and FUNDC1 in the TAC model or PE model.** A. Ten-week-old WT and *Fgf21 ^-/-^* mice were subjected to sham or TAC surgery for four weeks, and BNIP3L and FUNDC1 protein levels in the indicated groups. B. Neonatal rat cardiomyocytes were infected with si-NC or si-FGF21, and the protein levels of BNIP3L and FUNDC1 were measured in the indicated groups. All data represent mean ± SEM, and statistical significance was measured using the two-way ANOVA with the Bonferroni post-hoc test.


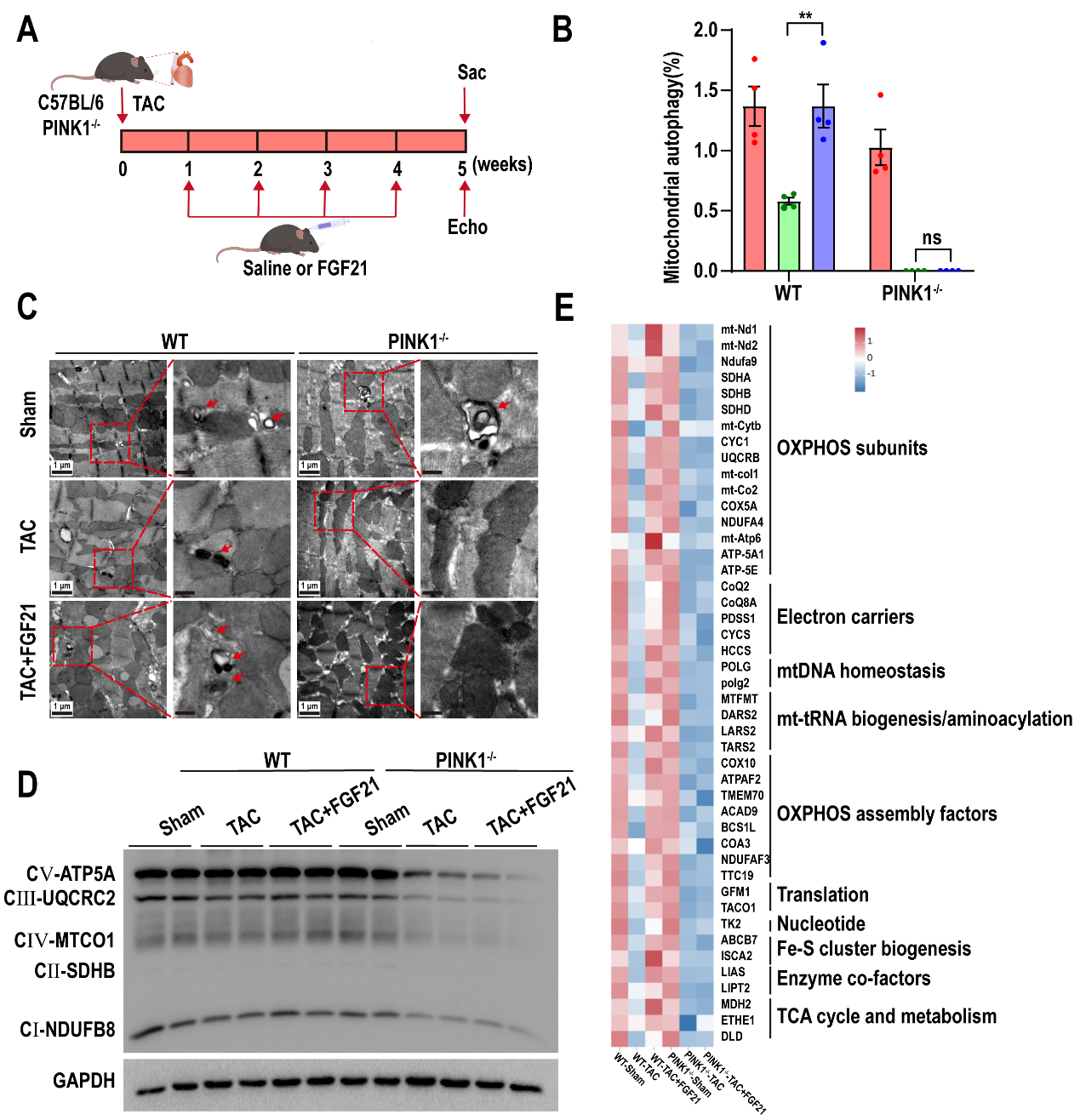


**Figure S4: Deletion of PINK1 inhibits mitophagy and energy metabolism compared with WT mice in the TAC model.** Ten-week-old WT and *Pink1^-/-^* mice underwent sham or TAC surgery. One week after the TAC, some TAC groups were subcutaneously injected with saline, and other TAC groups were subcutaneously injected with Fc-FGF21 fusion protein (Efruxifermin). The injection is once a week for four weeks. A. Schematic outline of animal experimental procedures. B. Quantitative analysis of the number of autophagosomes containing mitochondria for each group. C. Representative images from transmission electron microscopy, scale bar = 1 µm. D. Complex I, II, III, IV, and V protein levels. E. Relative factors of energy metabolism mRNA levels in the indicated groups. All data represent mean ± SEM, and statistical significance was measured using the two-way ANOVA with the Bonferroni post-hoc test.


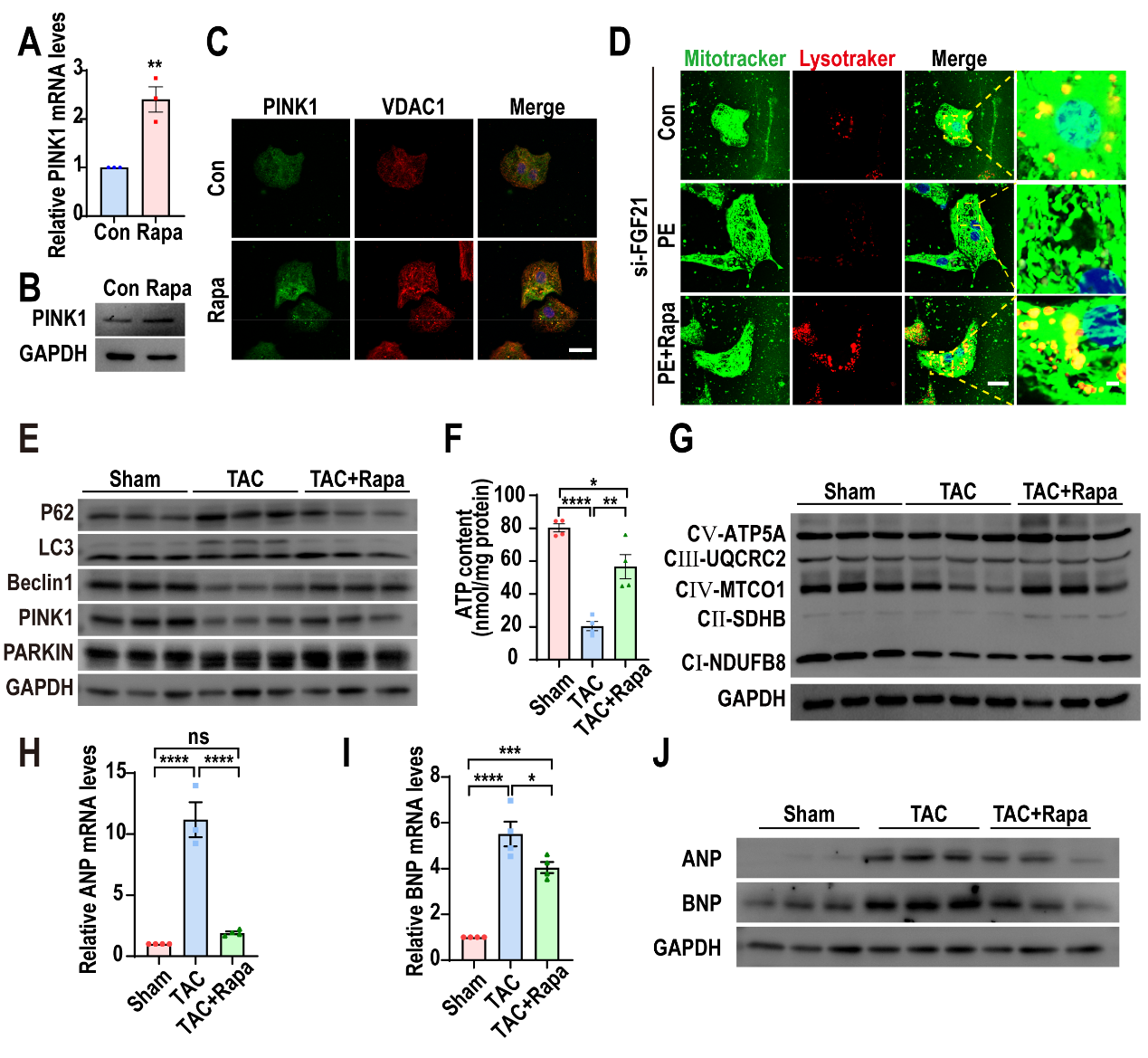


**Figure S5: Activation of the PINK1 via Rapa improves mitochondrial and cardiac function.** A**.** Neonatal rat cardiomyocytes were treated with Rapa, and the mRNA expression of PINK1 was detected by qPCR. B. Neonatal rat cardiomyocytes were treated with Rapa, and the protein of PINK1 was detected by Western blotting. C. Neonatal rat cardiomyocytes were treated with Rapa. Then, immunofluorescent staining was detected, scale bar = 20 μm. D. Neonatal rat cardiomyocytes were infected with si-FGF21, then treated with PE, and finally treated with Rapa. Mitochondria and lysosomes were stained with MitoTracker green dye and LysoTracker Deep Red dye, scale bar = 20 μm. E. P62, LC3, Beclin1, PINK1, PARKIN protein levels. F. ATP level in the indicated groups. G. Complex I, II, III, IV, and V protein levels. H, I. Relative ANP and BNP mRNA levels. J. ANP and BNP protein levels. All data represent mean ± SEM, and statistical significance was measured using the One-way ANOVA followed by Dunnett’s post-hoc test.


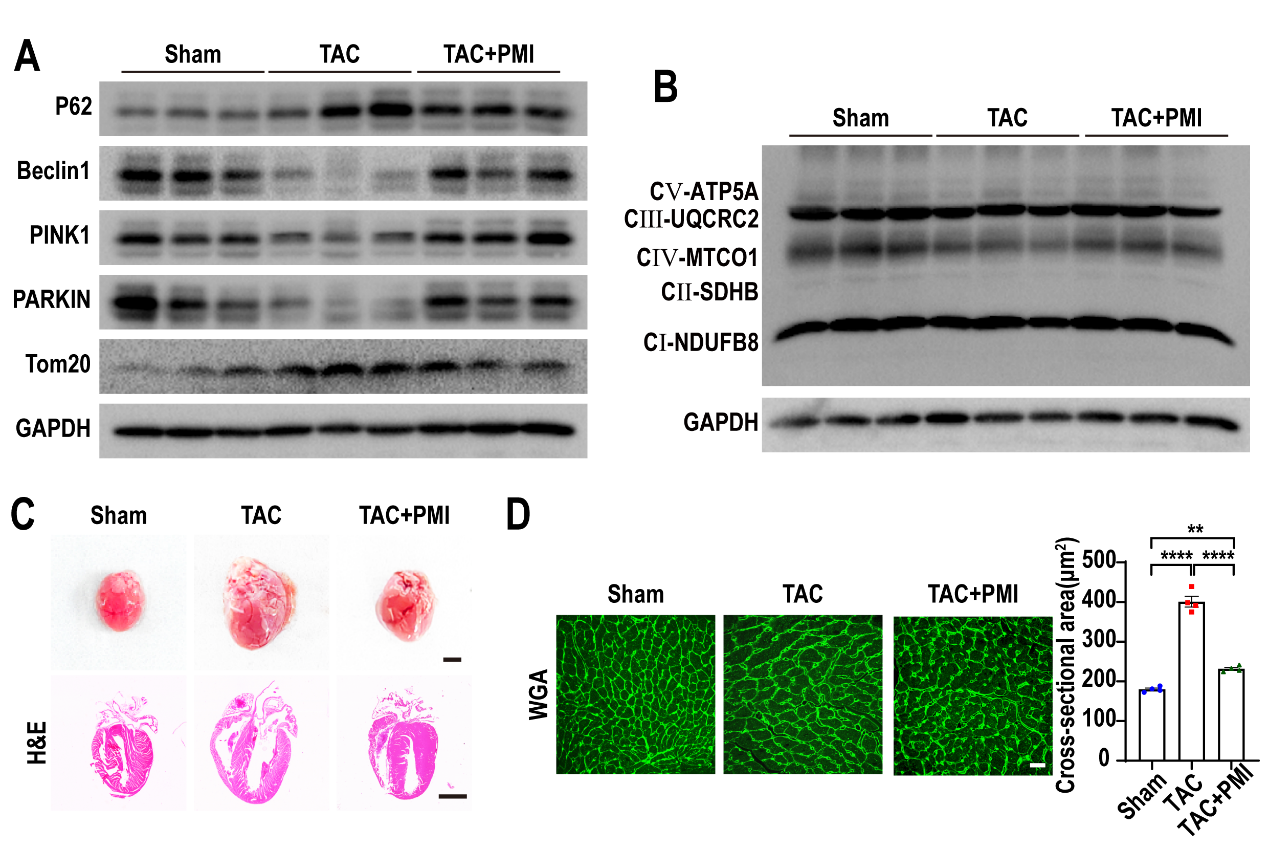


**Figure S6: Activation of mitophagy via PMI improves mitochondrial and cardiac function**. A. P62, Beclin1, PINK1, PARKIN, Tom20 protein levels. B. complex I, II, III, IV, and V protein levels. C. Ten-week-old *Fgf21^-/-^* mice were subjected to sham or TAC surgery. One week after the TAC, the sham group and TAC group were intraperitoneally injected with solvent, and the other TAC group was intraperitoneally injected with PMI for two weeks. Gross hearts, scale bar = 2 mm, and representative image of HE staining, scale bar = 2 mm (n = 4). D. Representative image of WGA immunofluorescent staining (n = 4), scale bar = 20 µm, quantitative analysis of cardiomyocyte cross-sectional area for each group. All data represent mean ± SEM, and statistical significance was measured using the One-way ANOVA followed by Dunnett’s post-hoc test.


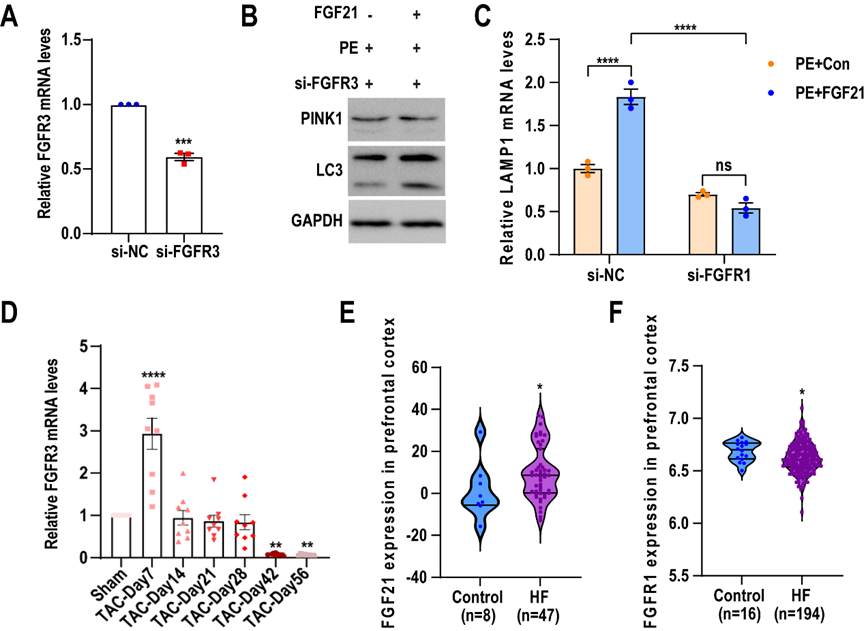


**Figure S7: The expression of FGF21, FGFR1, and FGFR3 in HF.** A**.** Relative FGFR3 mRNA level. B. Neonatal rat cardiomyocytes were infected with si-FGFR3, and then treated with control or 100 μM PE for 24 h, followed by vehicle or FGF21 protein. LC3 and PINK1 protein levels. C. Neonatal rat cardiomyocytes were infected with si-FGFR1, and then treated with control or 100 μM PE for 24 h, followed by vehicle or FGF21 protein. Relative LAMP1 mRNA levels. D. WT mice were taken from the TAC model at different times, relative to FGFR3 mRNA levels. E. Expression levels of FGF21 in the validation dataset GSE120895. F. Expression levels of FGFR1 in the validation dataset GSE5406. All data represent mean ± SEM, and statistical significance was measured using the One-way ANOVA followed by Dunnett’s post-hoc test.

Table S1 Primers for qPCR

| Gene name | Forward or 5' primer | Reverse or 3' primer |
| --- | --- | --- |
| ANP(mouse) | AGTGCGGTGTCCAACACAGAT | TCCTTGGCTGTTATCTTCGGTA |
| BNP(mouse) | GAGGTCACTCCTATCCTCTGG | GCCATTTCCTCCGACTTTTCTC |
| MYH7(mouse) | CAAAGGCAAGGCAAAGAAAG | TCACCCCTGGAGACTTTGTC |
| FGFR1(mouse) | CTGAAGGAGGGTCATCGAAT | GTCCAGGTCTTCCACCAACT |
| FGFR2(mouse) | CCTGCGGAGACAGGTTTCG | AACATCTTCACTGCCACGGT |
| FGFR3(mouse) | GCGTGGTGAGTTGGGCTCTA | ACCAGGAGGCTCGGAAGTAG |
| FGFR4(mouse) | TACTGGACACACCCCAACGCAT | GTACACCTTGCAGAGTAGCTCCA |
| FGF21(mouse) | GCATACCCCATCCCTGACTC | GACTTTCTGGACTGCGGTGT |
| GAPDH(mouse) | CGACTTCAACAGCAACTCCCACTCT | TGGGTGGTCCAGGGTTTCTTACTCCTT |
| ANP(Rat) | ATCTGATGGATTTCAAGAACC | CTCTGAGACGGGTTGACTTC |
| BNP(Rat) | TGACGGGCTGAGGTTGTTTT | ACACTGTGGCAAGTTTGTGC |
| PINK1(Rat) | GCAATGCCGCTGTGTATGAA | TCCCCATCTGCTCCCTTTGA |
| FGFR1(Rat) | CTGGCAGCGATACCACCTAC | GCCTACGGTTTGGTTTGGTG |
| FGFR3(Rat) | ACTGCAAGGTGTACAGCGAC | ACCTCTAGCTCCCTGTCGG |
| LAMP1(Rat) | CCACAGGATCAACCTTCCCC | ATGCTCTGGTCACAGTCGTG |
| GAPDH(Rat) | AGAGACAGCCGCATCTTCTT | GGGTTTCCCGTTGATGACCA |
